# AQUA-DUCT 1.0: structural and functional analysis of macromolecules from an intramolecular voids perspective

**DOI:** 10.1093/bioinformatics/btz946

**Published:** 2019-12-20

**Authors:** Tomasz Magdziarz, Karolina Mitusińska, Maria Bzówka, Agata Raczyńska, Agnieszka Stańczak, Michał Banas, Weronika Bagrowska, Artur Góra

**Affiliations:** Tunneling Group, Biotechnology Centre, Silesian University of Technology, 44-100 Gliwice, Poland

## Abstract

**Motivation:**

Tunnels, pores, channels, pockets and cavities contribute to proteins architecture and performance. However, analysis and characteristics of transportation pathways and internal binding cavities are performed separately. We aimed to provide universal tool for analysis of proteins integral interior with access to detailed information on the ligands transportation phenomena and binding preferences.

**Results:**

AQUA-DUCT version 1.0 is a comprehensive method for macromolecules analysis from the intramolecular voids perspective using small ligands as molecular probes. This version gives insight into several properties of macromolecules and facilitates protein engineering and drug design by the combination of the tracking and local mapping approach to small ligands.

**Availability and implementation:**

http://www.aquaduct.pl.

**Supplementary information:**

Supplementary data are available at *Bioinformatics* online.

## 1 Introduction

One of the most extensively used methods for the *in silico* study of macromolecules is molecular dynamics (MD) simulation. MD simulations have increased our knowledge of the conformational changes of proteins’ regulatory elements such as gates (Gora *et al.*, 2013) or loops (Kreß *et al.*, 2018). They improved our understanding of the role of water in protein folding and stability, in shaping enzyme activity and selectivity, or in drug design (Mondal *et al.*, 2017; Spyrakis *et al.*, 2017). Finally MD simulations enabled analysis of intramolecular voids, described as cavities (Stank *et al.*, 2016) and tunnels (Kingsley and Lill, 2015; Marques *et al.*, 2016), contributing to the macromolecules’ stability, functionality, activity and selectivity (Kokkonen *et al.*, 2019). More than 64% of enzymes are equipped with active sites buried inside the protein core (Pravda *et al.*, 2014), and investigation of the ligands’ entry pathways is considered as essential for future improvements in *de novo* designed enzymes (Huang *et al.*, 2016). However, the description of protein interior dynamics is not a trivial problem, since the commonly used sphere approximation fails to give an accurate description of asymmetric volumes and neglects the physicochemical properties of the interior—factors essential for the transportation of reagents (Kaushik *et al.*, 2018).

## 2 Materials and methods

**Fig. 1.**
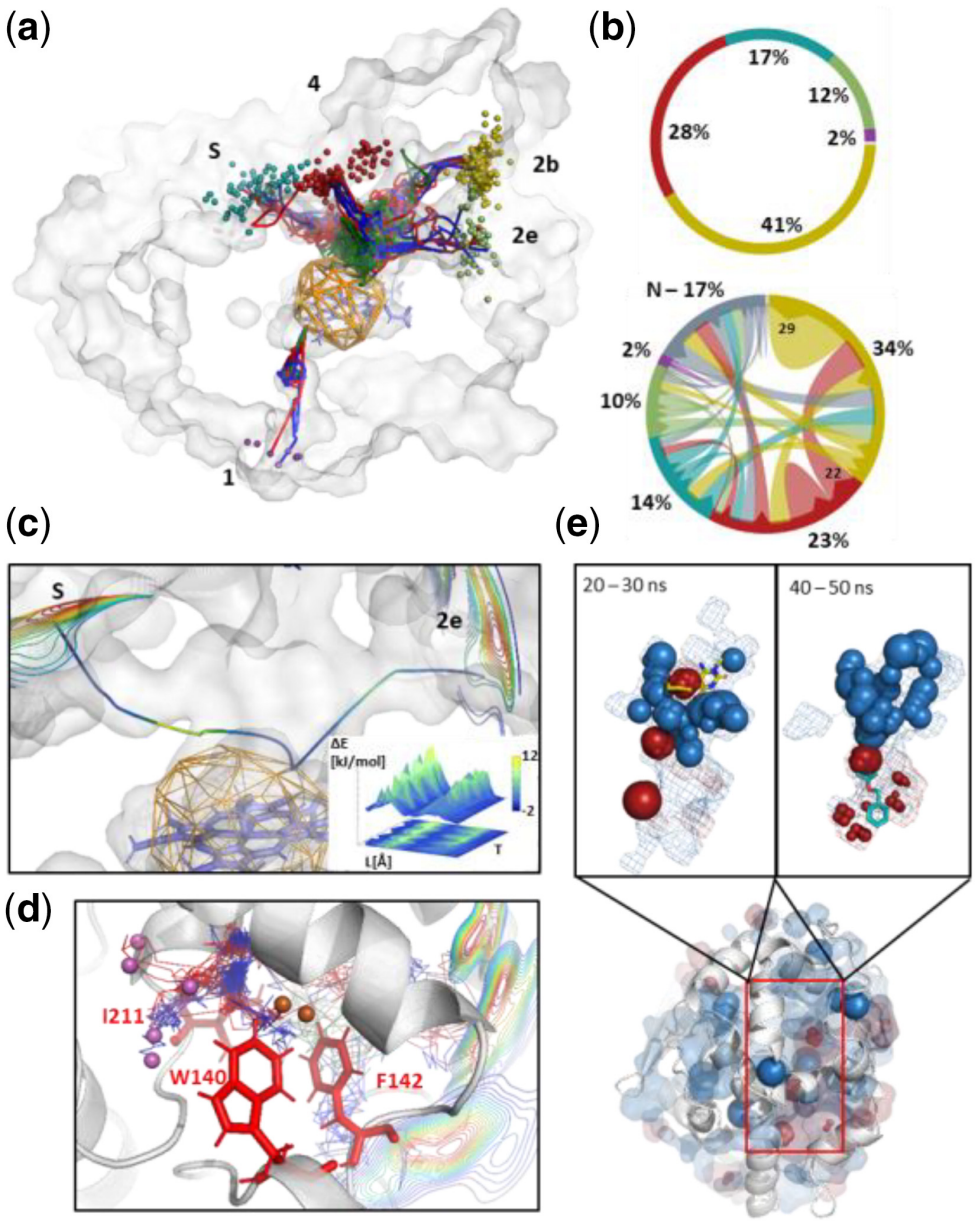
An example of AQUA-DUCT analysis. **(a)** Paths (lines) and entry/exit locations (small balls) of water molecules passing via cytochrome P450 3A4-binding cavity during 50-ns MD simulation. **(b)** Statistical data of tunnels entry utilization (upper) and flow between tunnel entries (lower part). Colors reflect ones used in (a). N indicates trajectories which start or end in protein interior. **(c)** Energy profile of water transportation between ‘2e’ and ‘s’ tunnel entries (shown as isolines) in cytochrome P450 3A4. The smoothed path colored according to energy scale, the energy profile calculated in 10-ns time-window. **(d)** Rare events analysis—detected leakage of water molecules in LinB haloalkane dehalogenase via pathway used for *de novo* tunnel design. Main tunnels entries are shown as isolines, leaking molecules as small balls. Modified residues indicated by red sticks. **(e)** Hot-spots of water (blue spheres) and DMSO (red spheres) identified by distribution analysis in human epoxide hydrolase in 50-ns simulation (middle panel). Inner pockets shown in surface representations. Overlap of detected hot-spots and inhibitors during analysis of different time frames of simulations are shown on bottom (3-[4-(benzyloxy)phenyl]propan-1-ol) and top (6-amino-1-methyl-5-(piperidin-1-yl)pyrimidine-2,4(1H,3H)-dione) panel. (Color version of this figure is available at *Bioinformatics* online.)

AQUA-DUCT 1.0 is an extension of the approach focused on molecules tracking (Magdziarz *et al.*, 2017). It goes beyond identification of the functionally relevant tunnels towards identification of structurally important residues and/or regions of macromolecules, approximation of free energy profiles of transportation pathways and an analysis of the evolution of the voids’ and hot-spots dynamics (Fig. 1 and Supplementary Fig. S1). It reverses the standard approach of describing the evolution of macromolecules’ dynamics through their atoms’ movement analysis and enables investigation of macromolecules from the perspective of ‘intramolecular voids’. To achieve this goal, we sample macromolecules’ dynamics employing small entities in simulations (most frequent water molecules, but also other co-solvent, ions or other ligands). They are used as specific ‘chemical probes’, and their trajectories (Supplementary Figs S2 and S3) and occupancies (Supplementary Fig. S4) are analyzed to discriminate between functionally relevant compartments and to overcome the limitations of geometrically based approaches.

### 2.1 Small molecules tracking analysis

AQUA-DUCT 1.0 allows not only to detect, describe and compare tunnels’ relevance and performance based on the number of molecules transported *via* a particular pathway (Fig. 1a and b), but also provides an approximation of transportation free energy profiles between pre-selected tunnels’ entries (Fig. 1c). The analysis of solvent molecules’ pathways allows for the identification of rare events which might correspond either to poorly sampled states, like *aquaduct* tunnel (W) in cytochrome P450 3A4 (Supplementary Fig. S5) or may suggest the localization of tunnels which can be designed *de novo* (Fig. 1d and Supplementary Fig. S6). Full statistical and quantitative analysis (Supplementary Fig. S7) is complemented by the visualization of raw and smoothed paths geometries (Supplementary Fig. S8), and the shape of ligands entry/exit areas (Supplementary Fig. S9).

### 2.2 Local-distribution analysis

The paths of molecules entering the protein interior can be structured and divided into distinct compartments corresponding to undisturbed passages and trapped molecules (Supplementary Fig. S2). The analysis of solvent trajectories can provide information about functionally relevant residues responsible for ligand trapping, which can vary depending on the tracked ligand (Supplementary Fig. S10). To simplify the identification of such residues, we calculate the local solvent distribution, which facilitates the detection of hot-spots, defined as compact volumes with high solvent occupancy (Supplementary Fig. S4). This approach can be used for the fast identification of functionally important residues (e.g. gates) or molecules (e.g. catalytic water molecules), the description of hydrophilic/hydrophobic regions in the protein core (Supplementary Figs S11 and S12) and also for drug design (Fig. 1e).

### 2.3 Modes

The AQUA-DUCT 1.0 provides four distinct modes of analysis (Supplementary Fig. S13). The *standard mode* is used for the routine analysis of a single MD simulation. The *sandwich mode* enables the parallel analysis of multiple runs of individual simulations with different topologies (approximation of a macroscopic picture of the analyzed molecule). The *time-window mode* allows the analysis of long trajectories in pre-defined time windows and thus facilitates the identification of equivalent or alternative states (Supplementary Fig. S12). Different and rare conformations can be correctly described with the *consolidator mode* (Supplementary Fig. S14). Pre-selected frames of the simulation can be merged together to provide a pre-treated trajectory with enhanced sampling of a rare event [e.g. substrate entry (Supplementary Fig. S14) or the rare opening of an alternative pathway Fig. 1d] and efficiently analyzed. The obtained data can be used for the alternative design of enhanced catalysts or new inhibitors, as well as used as high-quality preliminary data comparable with Markov model results.

## 3 Conclusions

Our method is able to analyze dynamic changes in the spatial distribution of the physicochemical properties with user-defined time-scales and resolution, and also with easy and fast insight into the geometry of macromolecules’ interiors and the approximation of transport energy barriers *via* particular pathways. The application of ‘ligands-tracking’ and ‘local-distribution’ approaches together with the introduction of a ‘chemical probe’ overcomes most of the limitations of currently available tools. The user receives direct access to information about the active site, potential hot-spots, functional residues, the network of internal transportation pathways and functional voids and cavities and benefits from modules that can facilitate the understanding of macromolecules, protein engineering and drug design. 

## Supplementary Material

btz946_Supplementary_Data
