# Supplementary material for: AQUA-DUCT 1.0: structural and functional analysis of macromolecules from an intramolecular voids perspective
Source: Bioinformatics. 2019 Dec 20;36(8):2599–601. doi: 10.1093/bioinformatics/btz946 (PMC7178393; doi:10.1093/bioinformatics/btz946)
Supplement: btz946_Supplementary_Data [file btz946_supplementary_data.pdf]

Supplementary data

AQUA-DUCT 1.0: structural and functional analysis of macromolecules from an intramolecular voids perspective.

Tomasz Magdziarz[1], Karolina Mitusińska[1], Maria Bzówka[1], Agata Raczyńska[1], Agnieszka Stańczak[1], Michał Banas[1], Weronika Bagrowska[1], Artur Góra[1]

[1]Tunneling Group, Biotechnology Centre, Silesian University of Technology, ul. Krzywoustego 8, 44-100 Gliwice, Poland

Contents

Implementation and configuration
Technical data of calculations by AQUA-DUCT

- **Valve**
- **Pond**

Case study

- **System selection and preparation**
- **Protein preparation**
- **MD simulations**

Supplementary note

- **Figure 1 full description**

Supplementary figures

- **Supplementary Fig. 1** Workflow of the AQUA-DUCT software.
- **Supplementary Fig. 2** Concept of the usage of small molecules tracking approach.
- **Supplementary Fig. 3** Examples of the analysis of MD simulations performed for different proteins.
- **Supplementary Fig. 4** Concept of the functionality of *pond* module.
- **Supplementary Fig. 5** An example of rare event detection in cytochrome P450 3A4.
- **Supplementary Fig. 6** An example of a rare event identification for protein modification analysis in *Bacillus megaterium* epoxide hydrolase.
- **Supplementary Fig. 7** Examples of the statistical and quantitative results presentation in AQUA-DUCT.
- **Supplementary Fig. 8** Different modes of paths representations.
- **Supplementary Fig. 9** Different modes of tracking molecules entry/exits area representations.
- **Supplementary Fig. 10** Hot-spots detection in AQUA-DUCT.
- **Supplementary Fig. 11** Detection of key amino acids by hot-spot module implemented in AQUA-DUCT.
- **Supplementary Fig. 12** Time window mode of AQUA-DUCT.
- **Supplementary Fig. 13** Overview of different modes of analysis provided by AQUA-DUCT.
- **Supplementary Fig. 14** An example of consolidator mode usage.
- **Supplementary Fig. 15** GUI – examples of the Graphical User Interface.
- **Supplementary Fig. 16** An example of *kraken* output file from analysis of sample data set.

Performance

Implementation and configuration

AQUA-DUCT is free software available at http://www.aquaduct.pl, licensed under GNU GPL v3. It facilitates the analysis of macromolecules based on tracking small molecules present in molecular dynamics simulations. It can be used for the analysis of the trajectories from most of molecular dynamics (MD) simulations packages. It uses MDAnalysis Python module (Michaud-Agrawal *et al.*, 2011) for reading, parsing and searching of MD trajectory data.

AQUA-DUCT comprises three calculation modules: *valve*, *pond*, and *kraken* (**Supplementary Fig. 1, Supplementary Fig. 2 and Supplementary Fig. 4**) working on top of *aquaduct* Python module. Parameters for calculations can be set by Graphical User Interface (GUI) using easy, normal, or expert mode (**Supplementary Fig. 15**). The results of the analysis can be visualized in PyMOL software (Schrödinger, 2015) and also further proceeded by *kraken* module (**Supplementary Fig. 16**).

AQUA-DUCT version 1.0 can be installed on Linux, Windows, macOS, OpenBSD systems.

Technical data of calculations by AQUA-DUCT

Valve

Basic definitions and tracking

The minimal setup for performing AQUA-DUCT calculations comprises of the MD trajectory and topology files, the specification of molecules to be traced (*Object*), and the definition of the spatial area in which tracking is performed (*Scope*) (**Supplementary Fig. 2**). Different types of molecules present in MD simulations can be tracked simultaneously (e.g., water, co-solvent, ligands, and other molecules). A list of the *traceable residues* consists of all defined molecules which were found in predefined *Object* and/or within predefined *Scope* and is created for all simulation frames. The *Object* definition represents the volume of focused interest (e.g., the active site of the protein, cofactor cavity, etc.) (**Supplementary Fig. 2**). The *Scope* defines the boundaries in which residues are traced and is usually defined as the interior of a convex hull of selected atoms of the macromolecule (**Supplementary Fig. 2**). The convex hull approach gives the advantage of being very fast; however, it is a coarse representation of the macromolecule's shape. To improve accuracy, AQUA-DUCT can run the so-called Auto-Barber procedure (Magdziarz *et al.*, 2017), which additionally trims the paths of the traced molecules to the approximated surface of the macromolecule or its selected parts (**Supplementary Fig. 2**), to mimic the protein surface, Auto-Barber can be set to all protein atoms using van der Waals radii correction.

Paths description

Each molecule from the list of *traceable residues* is represented as a single path that stores information about frames in which a molecule is in *Scope* or in *Object* including information about coordinates of its center of geometry. This raw paths' data is further transformed into two types of *Paths*: *Passing Paths* – traces of molecules that entered *Scope* but did not visit *Object* area, and *Object Paths* – traces of molecules that visited *Object* (**Supplementary Fig. 2**). Each *Object Path* comprises of up to four parts: i) *Incoming* – a path that leads from the point in which molecule enters the *Scope* to the point in which it enters the *Object* for the first time, ii) *Outgoing* – a path that leads from the point in which residue leaves the *Object* for the last time to the point in which it leaves the *Scope*, iii) *Object* – a path that leads from the point in which residue enters the *Object* for the first time to the point it leaves it for the last time; *Object* part spans between the last point of *Incoming* part and the first point on *Outgoing* part, iv) *Out of object* – within *Object* part of *Path* molecules can temporarily leave the *Object* area and stay in *Scope*; these parts are distinguished as *Out of object* paths. (**Supplementary Fig. 2**).

Paths smoothing
Visual inspection of paths can be greatly improved by smoothing. AQUA-DUCT implements several smoothing methods, most notably Savitzky-Golay filter (Savitzky and Golay, 1964), and moving window-based methods. Paths are smoothed in a way to keep information about time-step. The smoothed paths are recommended also for energy profile construction.

Inlets and paths clustering
Starting and ending points of paths can be located at the surface or inside of the macromolecule. Points located at the surface, i.e., *inlets*, correspond to tunnels exits and have a natural tendency to form groups. AQUA-DUCT allows for automatic clustering of paths based on the position of the *inlet* in space. Clusters of paths represent different potential transportation pathways between certain exits/entries areas and can be analyzed separately.
The clustering can be run in a recursive manner, including clusters merging or division, followed by optional *outliers* reclustering. The recommended method for clustering is *barber* which is based on the Auto-Barber procedure. Other methods are implemented with *sklearn.cluster* (Pedregosa *et al.*, 2011) Python module including: i) MeanShift, ii) DBSCAN, iii) AffinityPropagation, iv) KMeans, v) Birch methods. Selection of methods and available options (re-clustering, flexible clusters merging and splitting), facilitate proper clustering according to user needs.

Tunnel ends visualisation
The clustered *inlets* of the paths provide information about macromolecule's tunnels entry/exits. They can be visualised in two distinctive manners: i) as groups of *inlets* presenting in a simple way visualisation of the distribution of the endpoints in space or ii) as cluster areas approximated with kernel density estimation method (KDE) (Jones *et al.*, 2001) (**Supplementary Fig. 9**).

Master Paths
Clusters of paths that represent different potential transportation pathways (i.e. starting in one cluster of inlets (or interior – N) and ending in the second cluster of inlets (or in protein interior – N)) can be used to derive Master Paths i.e., paths that are averaged representation of all paths constituting a given pathway. AQUA-DUCT can generate several types of *Master Paths* with different smoothing settings (**Supplementary Fig. 8**). Smoothed *Master Paths* are recommended for energy profile calculation.

Pond

Pond component is an application that uses *aquaduct* module to perform further analysis of results from *valve*, including pockets analysis, hot-spots detection, and energy profile calculations (**Supplementary Fig. 4**).

Pockets
Pockets are calculated by analysis of paths found by *valve*. A regular grid is constructed spanning all paths. By default, the grid size is 1 Å and can be adjusted by the user. For each grid cell, the density of tracked molecules is calculated. Grid cells with nonzero density are used for pockets detection. Pockets can be partitioned into areas of a different overall distribution of traced molecules. By default, *pond* saves two types of pockets: i) *Inner pocket* – the part of the pocket for which densities are greater than global median value; this pocket represents an area that is easily accessible by traced molecules, ii) *Outer pocket* – the part that corresponds to the maximal possible space explored by all traced molecules. Optionally, the other values of the threshold for discrimination of the *Inner pocket* can be provided (e.g., % of the highest density value) (**Supplementary Fig. 12**).

Hot-spots detection
Further analysis of the distribution of densities in the grid allows selecting points of the highest local density (**Fig. 1e**, **Supplementary Fig. 10** and **Supplementary Fig. 11)**. They are considered as hot-spots, i.e., points of particular importance at which traced molecules are either attracted by favorable interactions with nearby amino acids or where they are trapped and stays for a considerably long time. In both cases, hot-spots can mark regions of particular importance for the macromolecule's functions.

Energy approximation
Pond can estimates energy profiles of user-defined paths (**Supplementary Fig. 4**) by using the calculated density of traced molecules. It becomes particularly useful and relevant when traced molecules include a solvent. Estimation of free energy is done according to Boltzmann's inversion. A similar method was used for energy approximation along ion channels (Rao *et al.*, 2017).
Following equation relates free energy with a density of molecules:

$$n(z) = C \cdot e^{\left(\frac{-E(z)}{kT}\right)}$$

Where z is a point in the space, *n(z)* is a density of molecules in point *z*, *C* is a normalization constant, *E* is free energy, *k* is Boltzmann's constant, and *T* is temperature.
One can easily transform the above equation to calculate energy:

$$E(z) = -kT\ln\left(n(z)\right) - kT\ln\left(C\right)$$

Term kT ln (C) does not depend on *z* and can be determined by the assumption that free energy in the bulk of traced molecules (solvent) is zero. This is done automatically in *pond* by determining the solvent only area of the system and calculation of the average density in that area.

Results analysis
Information about traced molecules and detected paths is provided in several ways including raw data tables and statistics (**Supplementary Fig. 7**), visualization (**Fig. 1** and **Supplementary Fig. 3**), or could be analysed automatically and plotted by *kraken* module (**Supplementary Fig. 16**).

Case study

Systems selection and preparation

P450 cytochrome CYP3A4b
The crystal structure of P450 cytochrome CYP3A4b (PDB ID: 2V0M) was downloaded from the Protein Data Bank. Missing residues were modelled by homology modelling using automodel class implemented in Modeller 9.14 (Sali and Blundell, 1993). The heme parameters were taken from Shahrokh et al. (Shahrokh *et al.*, 2012)

Human epoxide hydrolase
Two crystal structures of *Homo sapiens* soluble epoxide hydrolase (hsEH; PDB IDs: 1S8O and 4JNC) were downloaded from the Protein Data Bank. In the case of 1S8O the phosphatase domain was removed manually from the crystal structure. In the case of 4JNC the ligand was removed manually from the crystal structure.

Bacillus megaterium epoxide hydrolase
The crystal structure of *Bacillus megaterium* epoxide hydrolase structure (BmEH; PDB ID: 4NZZ) was downloaded from the Protein Data Bank and chain A was selected for analysis.

Solanum tuberosum epoxide hydrolase
The crystal structure of *Solanum tuberosum* epoxide hydrolase (StEH1; PDB ID: 2CJP) was downloaded from the Protein Data Bank. Chain A and the ligand were removed manually from the crystal structure.

Haloalkane dehalogenase LinB
The crystal structure of the wild type of haloalkane dehalogenase LinB from *Sphingomonas paucimobilis* UT26 (PDB ID: 1MJ5) was downloaded from the Protein Data Bank. Metal ions were removed manually from the crystal structure. This structure was used also for double mutant construction (LinB73, D147C, L177C) using DDG monomer from Rosetta (Kellogg *et al.*, 2011).

D-amino-acid oxidase
The crystal structure of human D-amino-acid oxidase (hDAAO; PDB ID: 2DU8) was downloaded from the Protein Data Bank. The ligand was manually removed and the FAD cofactor structure was retained. The parameters used for FAD were taken from Stuchebrukhov et al. (Stuchebrukov *et al.*, 2000). Chain A was selected for analysis.

Cyanothece sp. lipoxygenase 2
The crystal structure of *Cyanothece sp*. lipoxygenase 2 enzyme (CspLOX2; PDB ID: 5MED) was downloaded from the Protein Data Bank. The ligands were manually removed and the iron ion was retained. The parameters for the iron ion were taken from Li and Merz (Li and Merz, 2014). Chain A was selected for analysis.

Protein preparation

Common steps
The protonation states of titratable residues were determined using the H++ Server (Anandakrishnan *et al.*, 2012) at pH 6.5 (hsEH, BmEH, CspLOX2), 6.8 (StEH1), 7.5 (cytochrome), 8.3 (hDAAO) and 8.5 (LinB WT and LinB73 mutant). In the case of all soluble epoxide hydrolases, CYP3A4b and hDAAO the crystal water molecules were retained and a combination of the 3D-RISM (Kovalenko *et al.*, 2010) and Placevent (Sindhikara *et al.*, 2012) were applied to place water molecules inside the protein cavities. LEaP was used to add counterions and immerse all models in a box of TIP3P water molecules.

System preparation for cosolvent analysis of human soluble epoxide hydrolase
The Packmol software (Martinez *et al.*, 2009) was used to build the initial configuration file with: i) protein, water, and DMSO molecules, ii) protein, water, DMSO and methanol molecules.

System preparation for cosolvent analysis of Solanum tuberosum epoxide hydrolase
The Packmol software (Martinez *et al.*, 2009) was used to build the initial configuration file with: i) protein, water, and DMSO molecules, ii) protein, water and methanol molecules.

Cosolvent concentration
Two factors were taken into account for the selection of the number of co-solvent molecules. The number should be high enough to contribute in exchange with protein interior, and should be tolerated by protein (the enzyme should be still active in proposed co-solvent concentration). The largest contribution of cosolvent was for the system with DMSO (400) and methanol (600) and the calculated final concentration was 12.22% for DMSO and 7.51% for methanol. According to literature data (Stepankova *et al.*, 2013) even much higher concentrations of the organic solvent can be used.

System preparation for substrate entry analysis of haloalkane dehalogenase LinB
The substrate 1,2-dibromoethane was downloaded from the ChemSpider database (Royal Society of Chemistry, 2015). Force field parameters and libraries for ligand were generated using R. E. D. Server

(Vanquelef *et al.*, 2011) with Gaussian 09 (Frisch *et al.*, 2010) as QM program and RESP-A1 and AMBERFF10 as a charge model and force field, respectively. AddToBox program (Cerutti *et al.*, 2008) was used to randomly placed four substrates around protein at a distance of 5 Å from the protein and from each other. The procedure was run 10 times to obtain ten different models.

Final systems composition
The simulation systems consisted of: i) P450 cytochrome CYP3A4b in a truncated octahedral water box with TIP3P water (14733) molecules and 4 Cl^- ions, ii) the chain A of BmEH in a truncated octahedral water box with TIP3P water (8548) molecules and Na^+ ions (9), iii) hydrolase domain of hsEH immersed in a truncated octahedral consisting of a mixture of TIP3P water (10980) and DMSO (400) molecules, iv) hydrolase domain of hsEH (4JNC) immersed in a truncated octahedral consisting of a mixture of TIP3P water (11220), DMSO (400), and methanol (600) molecules v) the chain B of StEH1 in a truncated octahedral consisting of a mixture of TIP3P water (11047) and DMSO (400) molecules, vi) the chain B of StEH1 truncated in a octahedral consisting of a mixture of TIP3P water (11047) and methanol (800) molecules, vii) LinB mutant in oxidative conditions in a truncated octahedral consisting of a mixture of TIP3P water (11857) with Na^+ (18) and Cl^- (9), viii) LinB mutant in reductive conditions in a truncated octahedral consisting of a mixture of TIP3P water (11066) with Na^+ (17) and Cl^- (8), ix) LinB in a truncated octahedral consisting of a mixture of TIP3P water (from 37212 up to 47366, depending on substrate molecules' position) with 1,2-dibromoethane (4), x) chain A of human D-amino-acid oxidase structure with FAD cofactor in a truncated octahedral consisting of a mixture of TIP3P water (18040) with Na^+ ions (6) and xi) chain A of *Cyanothece sp.* lipoxygenase 2 with iron ion in a truncated octahedral consisting of TIP3P water (21331) and Na^+ ions (7).

MD simulation

General remarks
Concerning simulation length, for small proteins (<100 kDa) a 50 ns run is a minimum for system description, however, if large conformational changes are expected, the simulation need to be elongated. For short simulations size (less than 100 ns) we recommend sampling every 1 ps, for longer simulations it can be increased up to 5 ps. The sampling can be also modified for larger molecules (e.g. we have used 2 ps sampling for substrate entry analysis). AQUA-DUCT is independent of MD software, therefore we do not recommend any particular force fields or water model; each MD package has his own combination, that is routinely used for protein study.

Soluble epoxide hydrolase
AMBER14 (D.A. Case, 2014) was used to perform the simulation procedure using ff14SB force field (Maier *et al.*, 2015). The minimization procedure consisted of 2000 steps, involving 1000 steepest descent steps followed by 1000 steps of conjugate gradient energy minimization, with decreasing constraints on the protein backbone (500, 125 and 25 kcal*mol-1*Å-2) and a final minimization with no constraints of conjugate gradient energy minimization. Gradual heating was performed from 0 K to 300 K over 20 ps using a Langevin thermostat with a temperature coupling constant of 1.0 ps in a constant volume periodic box. Equilibration and production were run using the constant pressure periodic boundary conditions for 2 ns (5 ns for hsEH) with 1 fs time step and 50 ns (100 ns for CspLOX2) with a 2 fs time step, respectively. The constant temperature was maintained using the weak-coupling algorithm for 50 ns (100 ns for CspLOX2) of the production simulation time, with a temperature coupling constant of 1.0 ps. Long-range electrostatic interactions were modelled using the Particle Mesh Ewald method with a non-bonded cut-off of 10 Å and the SHAKE algorithm. The coordinates were saved at intervals of 1 ps.

LinB mutant in oxidative and reductive conditions
To run a 100 ns simulation of LinB mutant in reductive and oxidative conditions, the AMBER14 (D.A. Case, 2014) package was used. Gradual heating was performed from 0 K to 298 K over 20 ps using a

Langevin thermostat with a temperature coupling constant of 1.0 ps in a constant volume periodic box. The coordinates were saved at intervals of 2 ps. Remaining parameters were the same as for other systems.

LinB WT with substrates

In the case of MD simulations of substrate entry to haloalkane dehalogenase LinB the Amber 18 package (Case, 2018) was used, other parameters were the same as in LinB mutants simulations.

Supplementary Figure 1. Workflow of the AQUA-DUCT software.

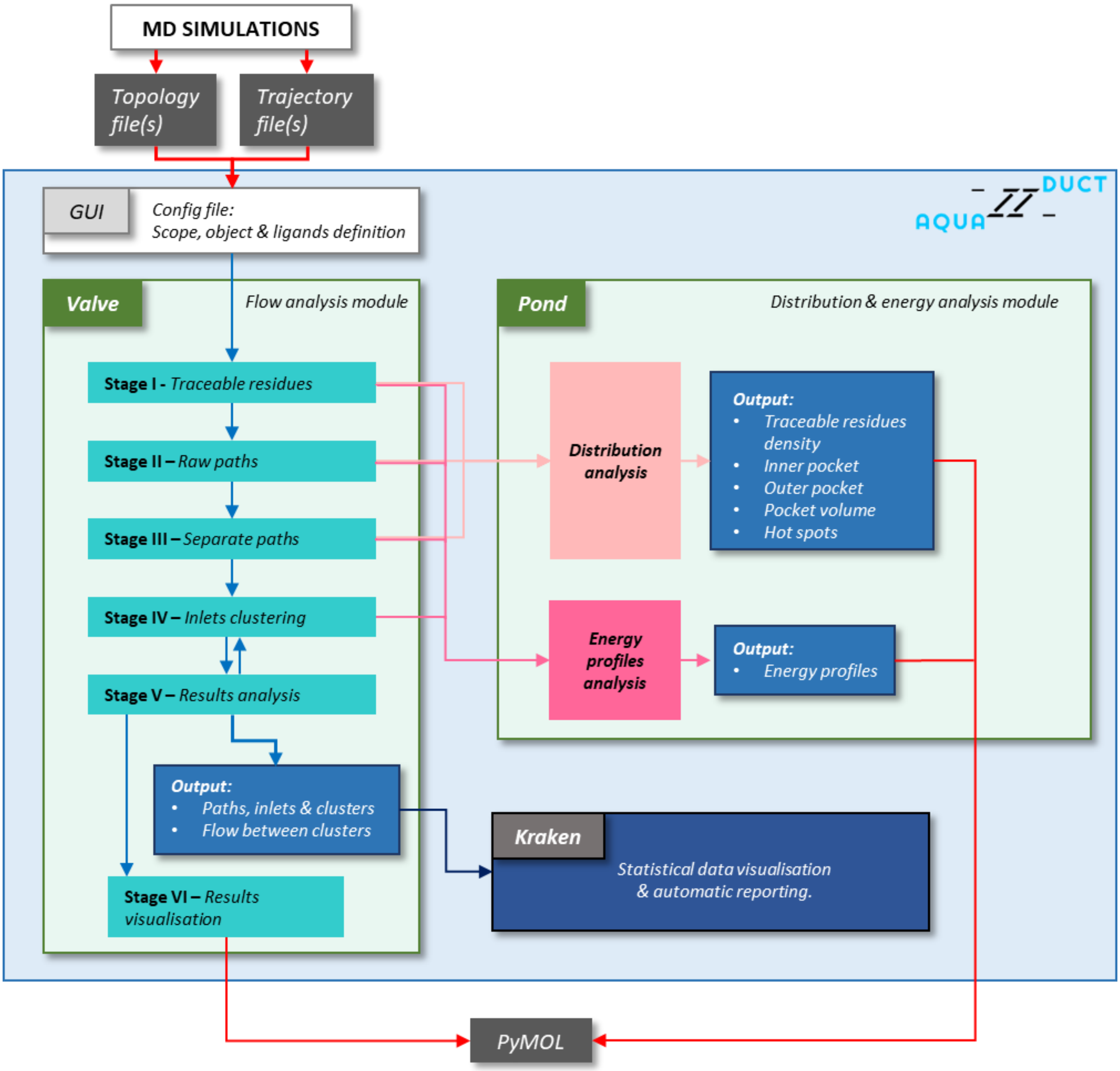

Supplementary Figure 2. Concept of the usage of small molecules tracking approach. (a) Schematic picture of the tracking-based approach. (b) Realization of the concept in practice. An example of two raw paths detected during analysis of cytochrome P450 3A4. Visualization in PyMOL (Schrödinger, 2015).

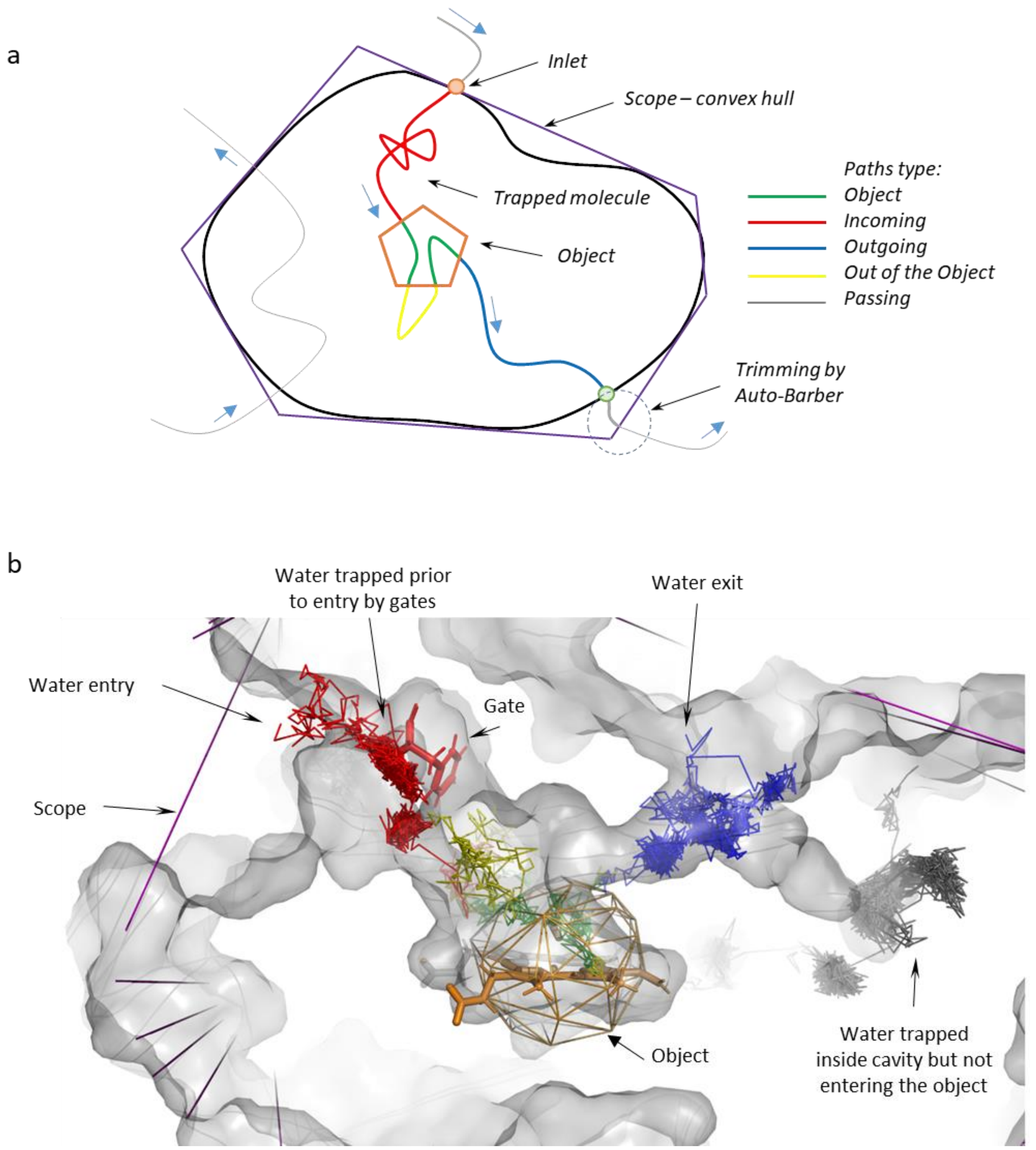

Supplementary Figure 3. Examples of the analysis of MD simulations performed for different proteins. (a) lipoxygenase, (b) human D-amino acid oxidase, and mutant of haloalkane dehalogenase LinB with introduced cysteine bridge (red stick) in oxidative (c) and reductive (d) conditions. Please note: i) single molecule pathway reflects oxygen tunnel position as suggested in work by Newie *et al.* (Newie *et al.*, 2017) ii) waters molecules detect not only main entrance to the active site (green and red) controlled by set of tyrosine residues (red sticks) (Subramanian *et al.*, 2018), but possibly hydrophobic tunnel (cyan) used for oxygen delivery to FAD cofactor (blue stick). The localization of the detected oxygen tunnel overlaps with those reported in structures from different species (Saam *et al.*, 2010; Rosini *et al.*, 2011). iii) Cysteine bridge is closing LinB structure (c) vs (d) and also is changing water flow distribution. The cysteine bridge mutant was used for further *de novo* tunnel design (Brezovsky *et al.*, 2016).

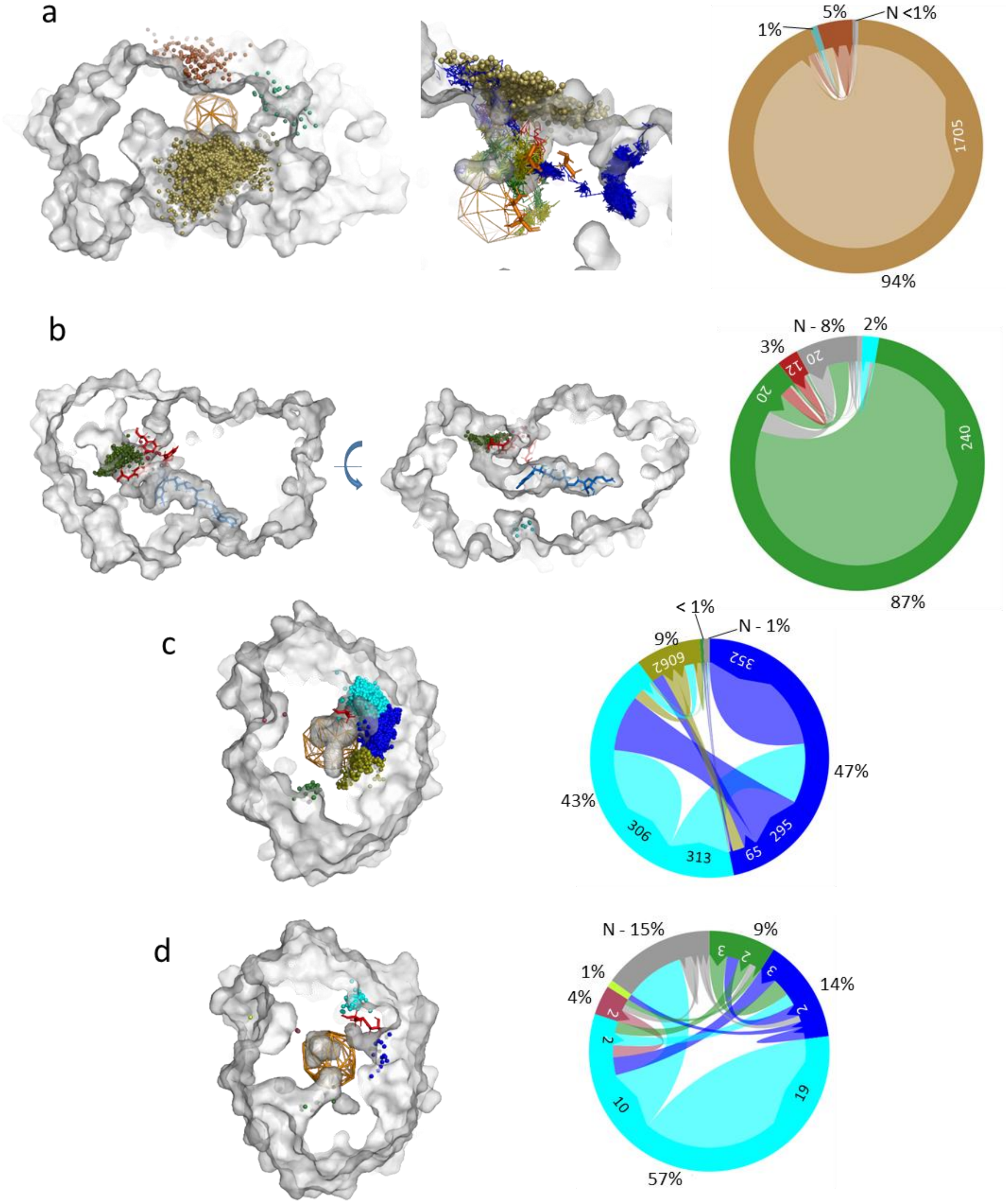

Supplementary Figure 4. Concept of the functionality of *pond* module. (a) Schematic picture of a cross-section of the protein (cytochrome) and grid colored according to detected water density. (b) Schematic picture of an enlarged active site area. The smoothed master path between two different tunnels entry is used for energy profile calculation. (c) Schematic picture of the calculated energy profile. (d) Schematic picture of an enlarged active site area and grid used for hot-spots calculation. (e) Schematic picture of density distribution. The cut-off position is calculated automatically based on the histogram constructed from all available data. (f) Schematic picture of final results of hot-spots identification, the blue sphere radius corresponds to density value.

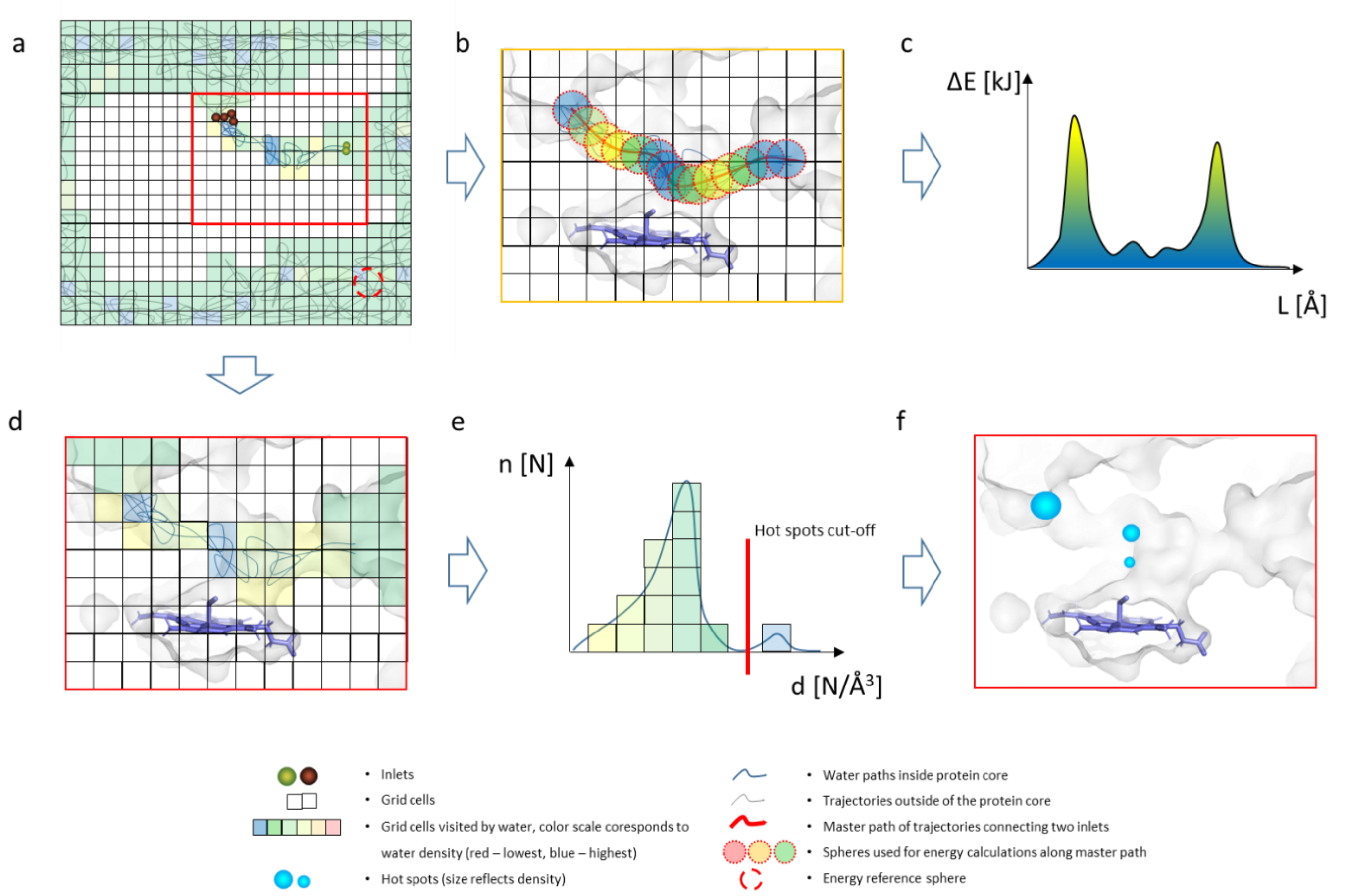

Supplementary Figure 5. An example of rare event detection. Single water molecule leakage *via aquaduct* tunnel (W) detected during 50 ns simulations of cytochrome CYP3A4. According to work done by Fishelovitch *et al.* (Fishelovitch *et al.*, 2010), the *aquaduct* tunnel is opened upon R375 (orange stick) side chain rotation and is facilitated by the FMN domain of cytochrome P450 reductase binding.

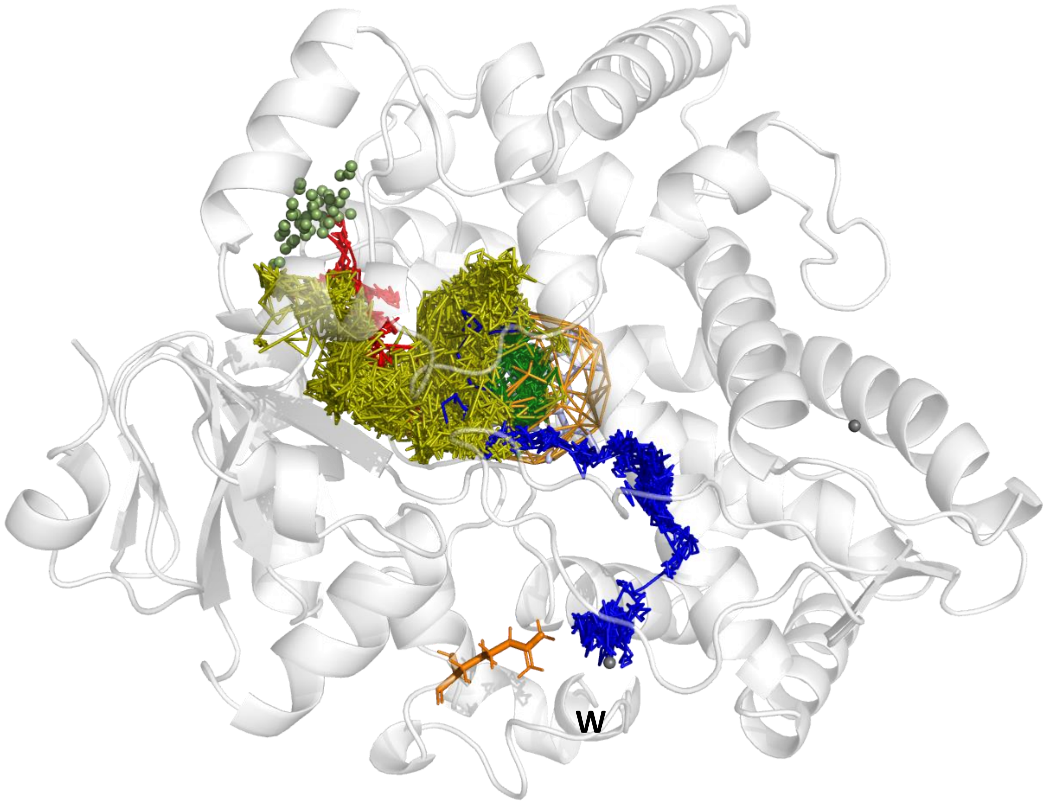

Supplementary Figure 6. An example of a rare event identification for protein modification analysis in *Bacillus megaterium* epoxide hydrolase. The analysis of water flow visiting active site cavity enabled the detection of a single water molecule pathway close to F128 residue. This phenylalanine residue was selected and mutated by Serrano-Hervás *et al.* (Serrano-Hervás *et al.*, 2018) to alanine to open the possibility to convert bulky substrates.

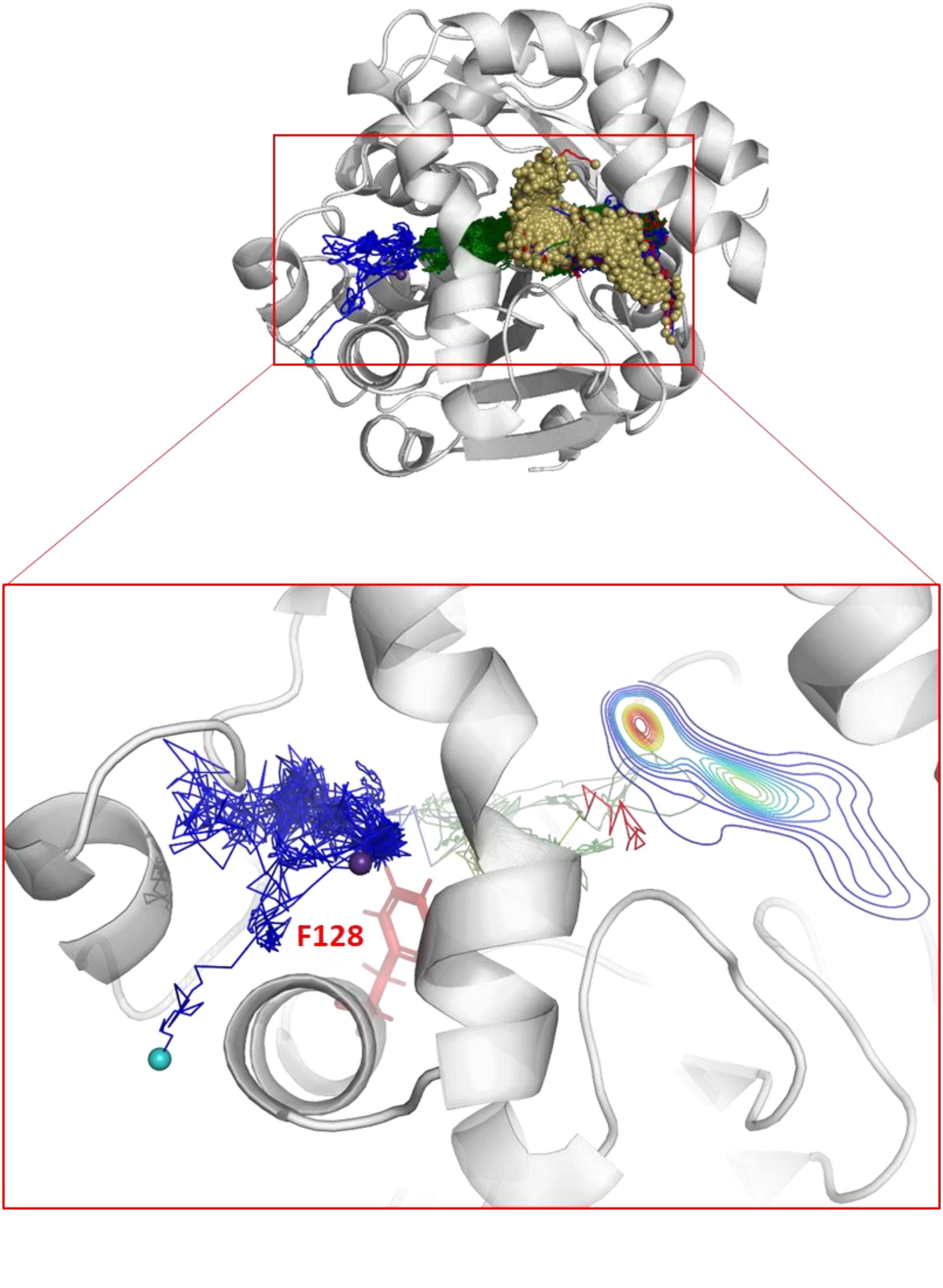

Supplementary Figure 7. Examples of the statistical and quantitative raw results presentation in AQUA-DUCT. Statistical and quantitative results obtained with AQUA-DUCT consist of a set of tables. The following shortcuts are used in all tables: *Nr* – cluster indexes, *Cluster* – respective AQUA-DUCT ID. All found outliers are gathered into cluster 0.

Table a) The summary of inlets that form individual clusters. *Size* – total number of inlets in a specific cluster, *INCOMING* and *OUTGOING* – number of inlets at the beginning and end of the detected paths, respectively.

Table b) The summary of cluster areas. The values in each column represent the area of the percentage ratio of inlets. D100 stands for the area of all inlets in a particular cluster, D95 - an area of 95% of inlets in particular clusters, etc.

Table c) The statistics of probabilities of separate paths transfers. *IN-OUT* – number of paths that both enter and leave the *Scope* by defined cluster, *diff* – number of paths that either 1) enter the *Scope* by this cluster but leave the *Scope* by another cluster, or 2) enter the *Scope* by another cluster but leave the *Scope* by this cluster, *N* – number of paths that either 1) enter the *Scope* by this cluster and stay in the *Object*, or 2) leave the *Scope* by this cluster after staying in the *Object*, *IN-OUT_prob*, *diff_prob*, and *N_prob* summarize probabilities of events listed in columns *IN-OUT*, *diff*, and *N*, respectively.

Table d) and e) The statistics of paths mean lengths in [Å] (d) and [number of frames] (e) of transfers. *X->Obj* – mean length of separate paths leading from this cluster to the *Object*, *Obj->X* – mean length of separate paths leading from the *Object* to this cluster, *p-value* – p-value of t-test of comparing *X->Obj* and *Obj->X* results, *X->ObjMin* – the minimal length of separate paths leading from this cluster to the *Object*, *ObjMinID* – ID of separate path for which *X->ObjMin* was calculated. *Obj->XMin* – the minimal length of separate paths leading from the *Object* to this cluster; *Obj->XMinID* – the ID of a separate path for which *Obj->XMin* was calculated.

Table f) and g) The summary of mean lengths in [Å] (f) and [number of frames] (g) of paths according to the Cluster Types (*CType*). Cluster Type classifies separated paths according to information about ID of the cluster in which tracked molecule entered the *Scope* and ID of the cluster in which it left the *Scope*, separated by ':'. *Size* – number of separate paths belonging to respective Cluster Type; *Tot* – average total length of paths with calculated standard deviation (*TotStd*). *Inp*, *Obj* and *Out* – average total length of Incoming, *Object* and Outgoing part of paths respectively with calculated standard deviation (*InpStd*, *ObjStd*, and *OutStd*); '*nan*' informs about lack of the particular part of the path.

Table h) The list of all separate paths and their properties. *RES* – residue name; *BeginF* and *EndF* – the number of the frame in which the path begins and ends; *InpF*, *ObjF* and *OutF* – the total number of the frames in which path is in Incoming, *Object* and Outgoing part respectively; *ObjFS* – the number of the frames in which path is strictly in *Object* part; *TotL*, *InpL*, *ObjL* and *OutL* – the information about length of the full path and the length of Incoming part, Object part and Outgoing part respectively in [Å]; *TotS* – average step of full path together with calculated standard deviation (*TotStdS*); *InpS*, *ObjS* and *OutS* – average step of Incoming, *Object* and Outgoing part respectively with calculated standard deviation (*InpStdS*, *ObjStdS* and *OutStdS*); *CType* – Cluster Type of each separate path; '*nan*' informs about lack of the particular part of the path.

a)

Clusters summary - inlets

Nr	Cluster	Size	INCOMING	OUTGOING
1	3	7	3	4
2	4	1	0	1
3	5	1	0	1
4	6	63	34	29

b)

Clusters summary - areas

Nr	Cluster	D100	D95	D90	D80	D70	D60	D50
1	3	30.98	20.21	18.49	16.02	13.67	10.83	8.53
4	6	117.87	108.21	100.09	87.18	73.76	58.09	44.53

c)

Clusters statistics (of paths) probabilities of transfers

Nr	Cluster	IN-OUT	diff	N	IN-OUT_prob	diff_prob	N_prob
1	3	2	0	3	0.40	0.00	0.60
2	4	0	1	0	0.00	1.00	0.00
3	5	0	0	1	0.00	0.00	1.00
4	6	6	38	13	0.11	0.67	0.23

d)

Clusters statistics (of paths) mean lengths of transfers

Nr	Cluster	X->Obj	Obj->X	p-value	X->ObjMin	X->ObjMinID	Obj->XMin	Obj->XMinID
1	3	6728.0	3550.6	0.1635	5198.1	0:5514:0	614.8	0:5514:0
2	4	nan	1943.5	nan	inf	None	1943.5	0:14276:0
3	5	nan	7601.7	nan	inf	None	7601.7	0:571:0
4	6	1899.4	1123.2	0.0477	142.1	0:3701:0	151.6	0:3554:0

e)

Clusters statistics (of paths) mean frames numbers of transfers

Nr	Cluster	X->Obj	Obj->X	p-value	X->ObjMin	X->ObjMinID	Obj->XMin	Obj->XMinID
1	3	9126.3	5009.8	0.2256	6575.0	0:5514:0	707.0	0:5514:0
2	4	nan	2229.0	nan	inf	None	2229.0	0:14276:0
3	5	nan	8085.0	nan	inf	None	8085.0	0:571:0
4	6	2494.1	1413.6	0.0368	160.0	0:3701:0	149.0	0:3554:0

f)

Separate paths clusters types summary - mean lengths of paths

Nr	CType	Size	Size%	Tot	TotStd	Inp	InpStd	Obj	ObjStd	Out	OutStd
1	8:8	29	12.78	7295.3	6279.38	1409.2	1202.42	4664.1	5650.85	1222.1	938.61
2	7:8	22	9.69	7873.1	4952.82	512.1	449.40	5178.5	4837.03	2182.5	1822.33
3	7:7	15	6.61	5878.4	4605.49	612.6	514.53	4710.6	4774.56	555.2	271.14
4	8:7	14	6.17	7996.7	5208.21	1330.4	939.68	6017.7	5185.46	648.7	502.39

g)

Separate paths clusters types summary - mean number of frames of paths

Nr	CType	Size	Size%	Tot	TotStd	Inp	InpStd	Obj	ObjStd	Out	OutStd
1	8:8	29	12.78	7764.9	6845.26	1642.8	1477.36	4688.7	6039.63	1433.5	1183.44
2	7:8	22	9.69	8382.1	5078.73	563.6	493.68	5136.5	4851.74	2682.0	2380.45
3	7:7	15	6.61	5967.7	4474.81	710.7	612.32	4610.0	4700.90	647.0	351.70
4	8:7	14	6.17	8035.9	5374.82	1520.6	1125.27	5772.1	5315.40	743.1	706.16

h[1])

List of separate paths and properties

Nr	ID	RES	BeginF	InpF	ObjF	ObjFS	OutF	EndF	TotL	InpL	ObjL	OutL
1	0:478:0	WAT	0	0	7103	1336	2039	9141	8146.8	nan	6472.7	1674.1
2	0:479:0	WAT	0	0	839	818	1779	2617	2499.9	nan	853.1	1646.8
3	0:480:0	WAT	0	0	5949	5790	468	6416	3864.7	nan	3505.8	358.9
4	0:481:0	WAT	0	0	2491	2298	545	3035	3221.9	nan	2677.2	544.7

h[2])

TotS	TotStdS	InpS	InpStdS	ObjS	ObjStdS	OutS	OutStdS	CType
0.89	0.531	nan	nan	0.91	0.549	0.82	0.453	N:6
0.96	0.527	nan	nan	1.02	0.535	0.93	0.520	N:8
0.60	0.352	nan	nan	0.59	0.346	0.77	0.390	N:6
1.06	0.607	nan	nan	1.07	0.613	1.00	0.574	N:7

Supplementary Figure 8. Different modes of paths representations: (a) raw, (b) raw master path, (c) smoothed path, (d) smoothed master path, (e) an example of a raw single path, (f) an example of a smoothed single path. For picture clarity, only paths between two tunnels entries (S – cyan, and 2b – sand) of cytochrome CYP34A are shown. Heme is shown in stick representation, protein as surface.

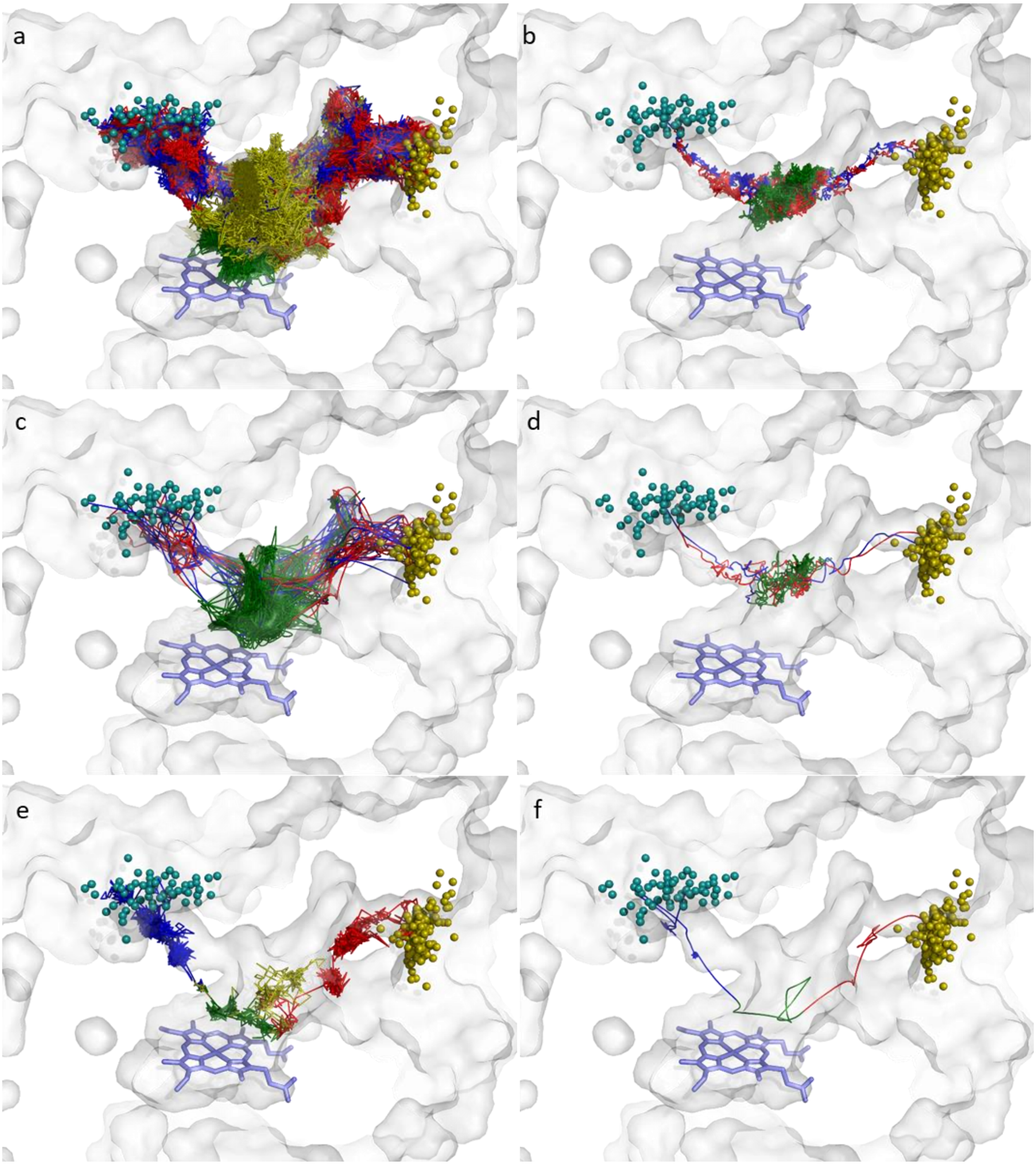

Supplementary Figure 9. Different modes of tracking molecules entry/exits area representations. (a) General view on *Bacillus megaterium* epoxide hydrolase with an entry tunnel located between cap (blue) and main (light grey) domain. Close-up on tunnel entrance indicated by water inlets (b) or cluster areas (c). Inlets (sand spheres) represent locations of water molecules entries/exits, the cluster areas are calculated with kernel density estimation method and provide information about the probability of entry/exit event in particular location. The isolines surround regions that cover particular percentage of inlets. Please note the large asymmetry of the shape of the tunnel entrance.

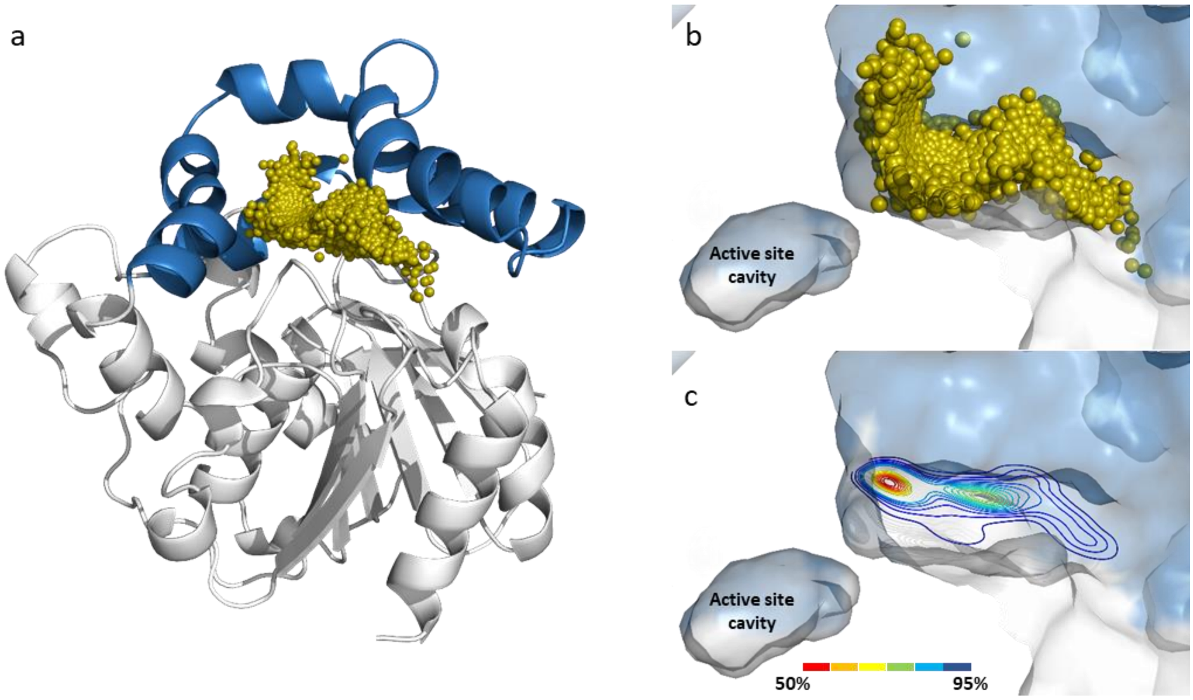

Supplementary Figure 10. Hot-spots detection in AQUA-DUCT - modes. Two modes of hot-spots detection implemented in AQUA-DUCT shown during analysis of 50 ns MD simulations of *Solanum tuberosum* epoxide hydrolase with different cosolvents. First and third rows (a-b; e-f) show hot-spots detected in presence of (a-b) DMSO (red dots) and water (blue dots) and (e-f) methanol (magenta dots) and water (blue dots). Hot-spots on the left panel were detected based on the molecules paths that visited the *Object*, whereas hot-spots on the right panel were detected based on the paths that entered the *Scope*. Second and fourth rows (c-d; g-h) present radial distribution of water and cosolvents as a function of distance from the active site for: c) DMSO and d) water, and g) methanol and h) water in respective environments.

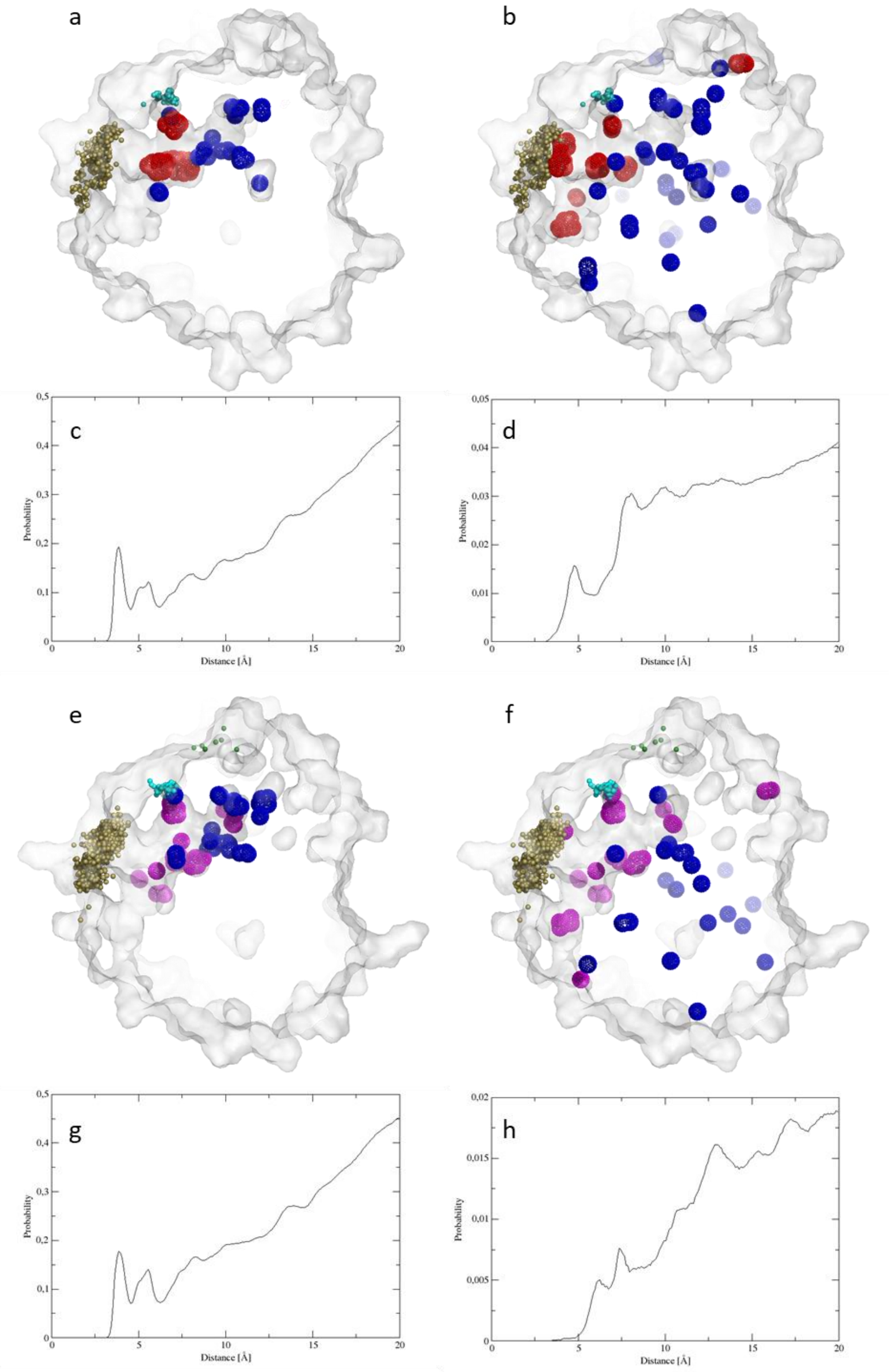

Supplementary Figure 11. Detection of key amino acids by hot-spot module implemented in AQUA-DUCT. Hot-spots detected during analysis of 50 ns long MD simulations of epoxide hydrolase from *Solanum tuberosum* provides information about residues important for catalytic activity (active site residues – red, and catalytic water stabilising residue – cyan), binding cavity shape (blue), gating residues controlling access to active site (orange and green) and potentially important residues in main tunnel (black) (Mitusińska *et al.*, 2018).

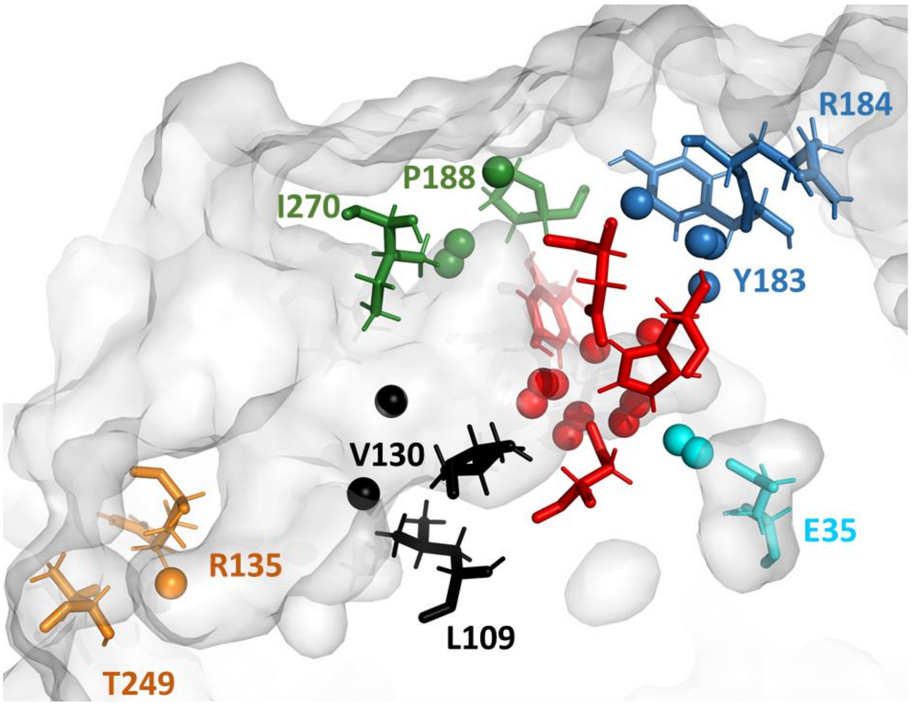

Supplementary Figure 12. Time window mode of AQUA-DUCT. Investigation of the time evolution of the solvent-accessible volume during 50 ns MD simulations of human soluble epoxide hydrolase immersed in a mixture of water DMSO and methanol. The first model in the picture shows a general distribution of inlets. The differences in solvent-accessible volume distribution facilitates detection of distinct protein conformations.

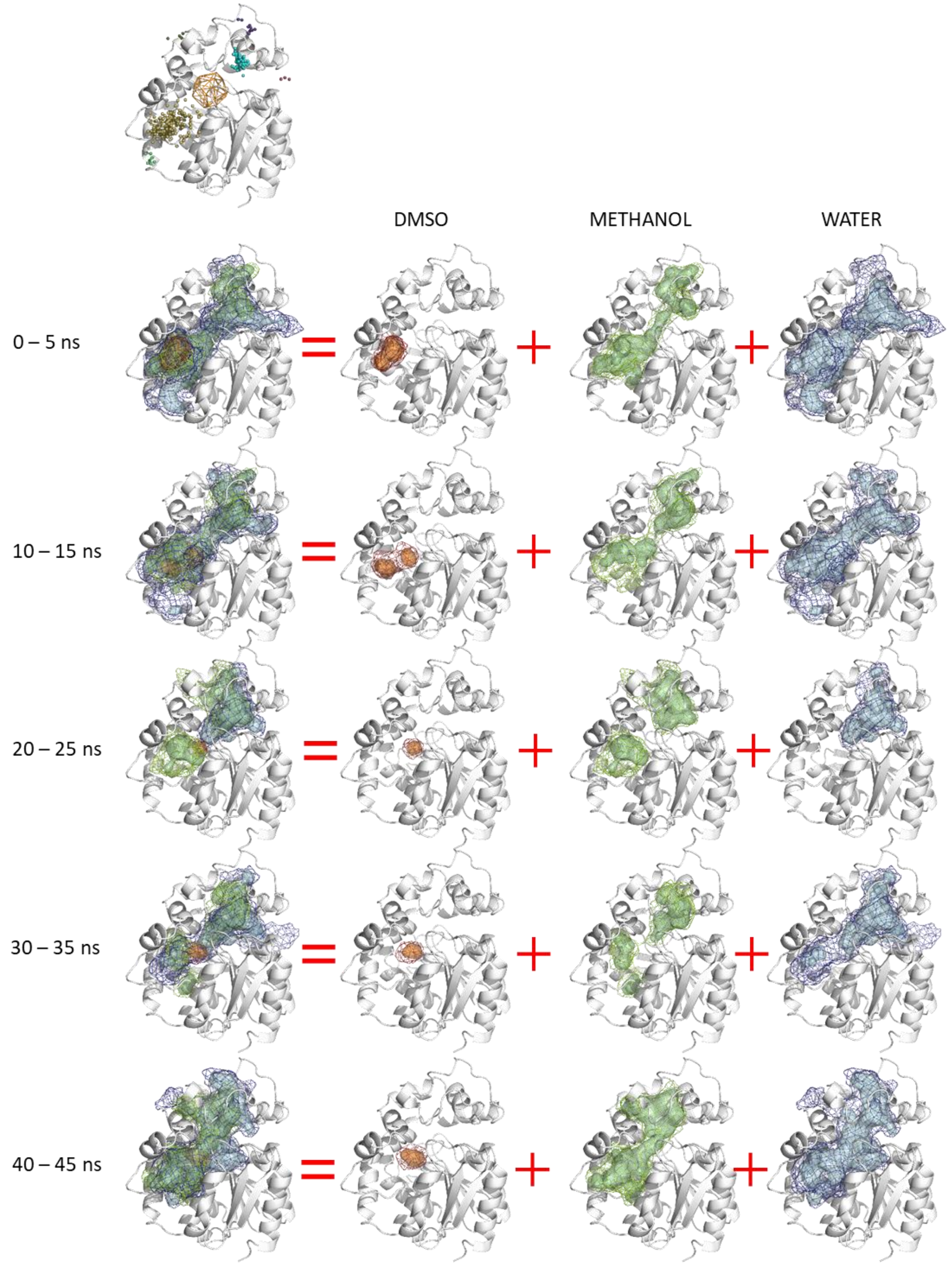

Supplementary Figure 13. Overview of different modes of analysis provided by AQUA-DUCT. The schematic information on required input (single or multiple trajectory files, or multiple fragments of MD trajectory runs), performed analysis and possible results in each mode are provided.

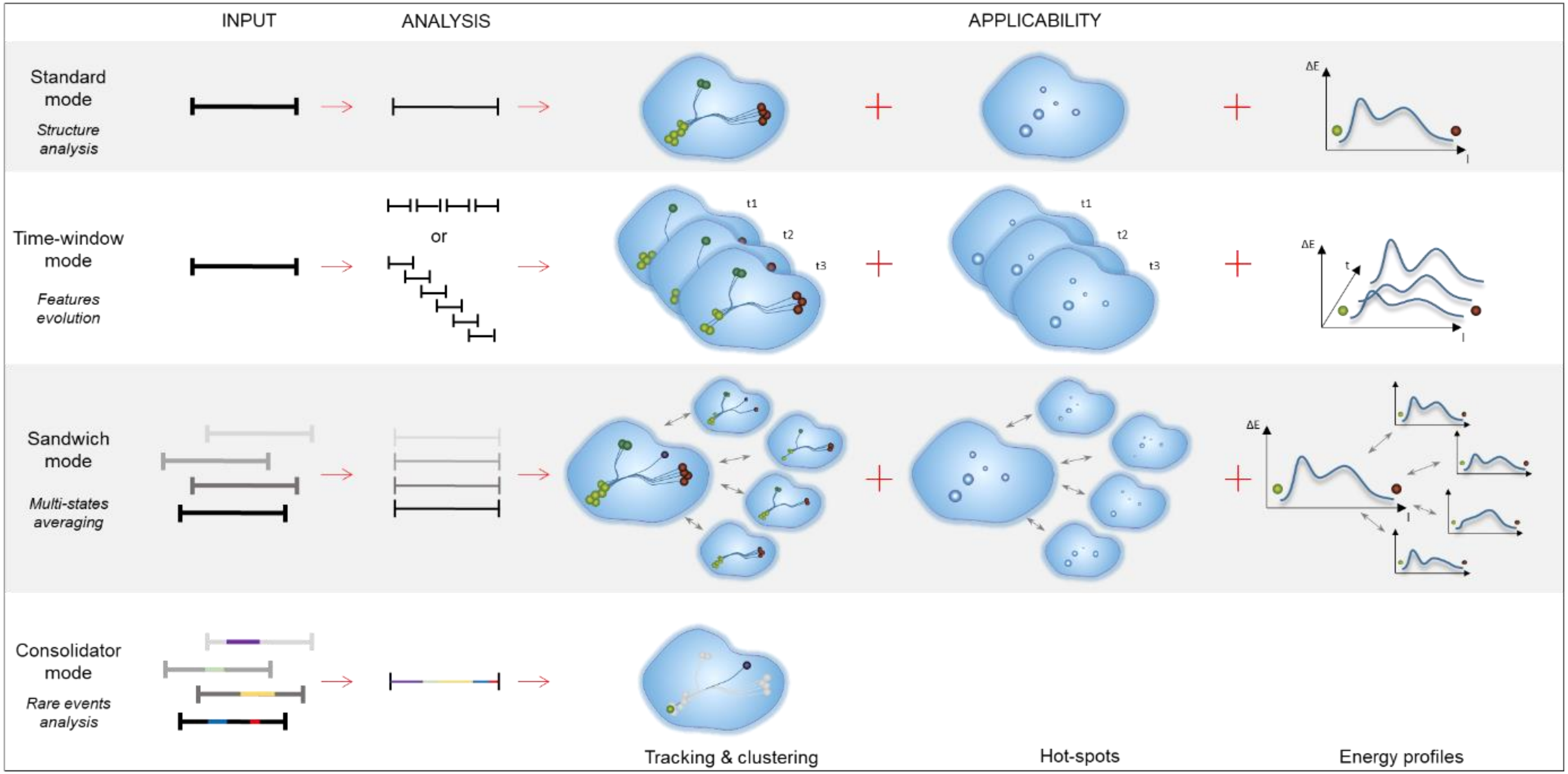

Supplementary Figure 14. An example of consolidator mode usage. (a) The distance between active site residues and substrates (4 molecules of 1,2 dibromoethane randomly distributed in the surrounding) is used for the pre-selection of frames in which substrate molecules were detected in protein core. Preselected frames are merged together as an input file for AQUA-DUCT. (b) Visualization of trajectories of all substrates from merged trajectories. (c) and (d) Two selected trajectories. The analysis of substrates entry suggests that substrate entry can be controlled by different residues than product exit. Please note, that 1,2 dibromoethane molecules are trapped before tunnels entry by L148 and V173 side chains (magenta), and their exit from active site cavity is blocked by other pairs of residues (D146, L177) (blue). The latter ones are known as gates of product release (Biedermannová *et al.*, 2012). Active site residues are shown in red.

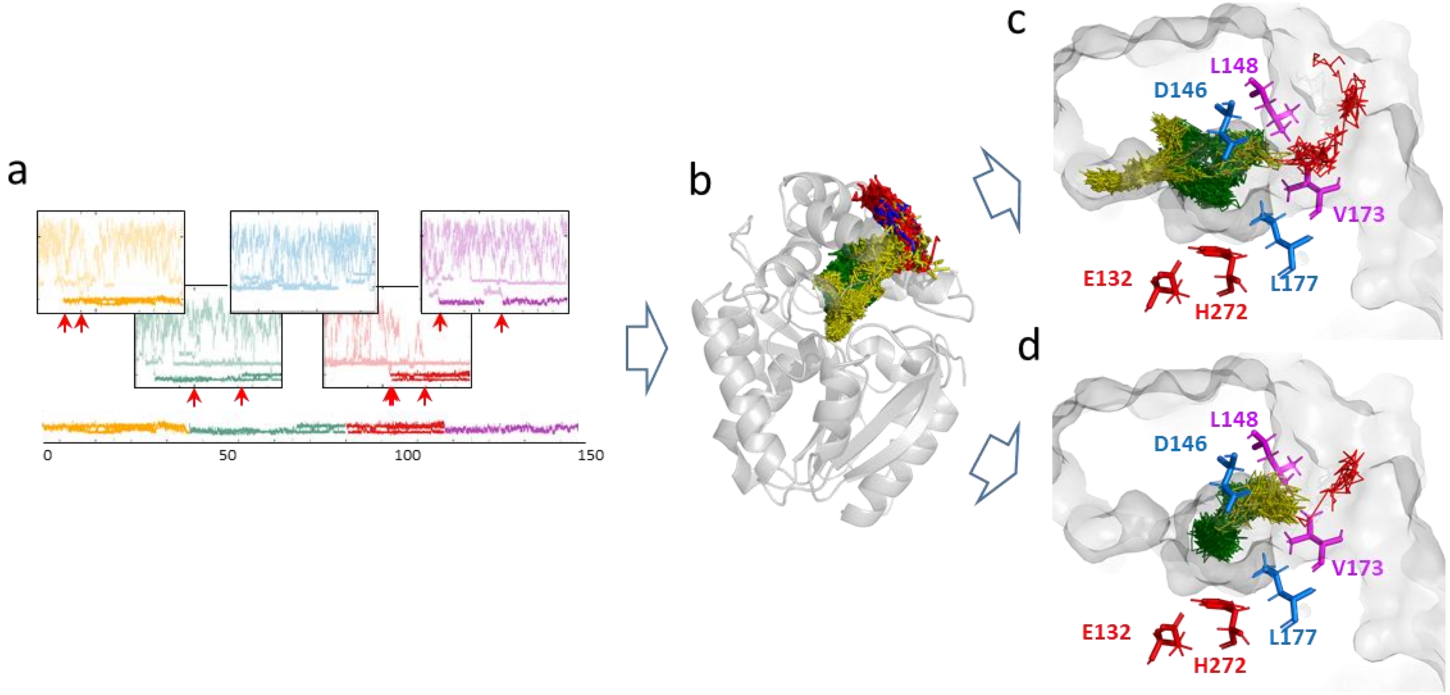

Supplementary Figure 15. GUI – examples of the Graphical User Interface in (a) Easy, (b) Normal and (c) Expert mode.

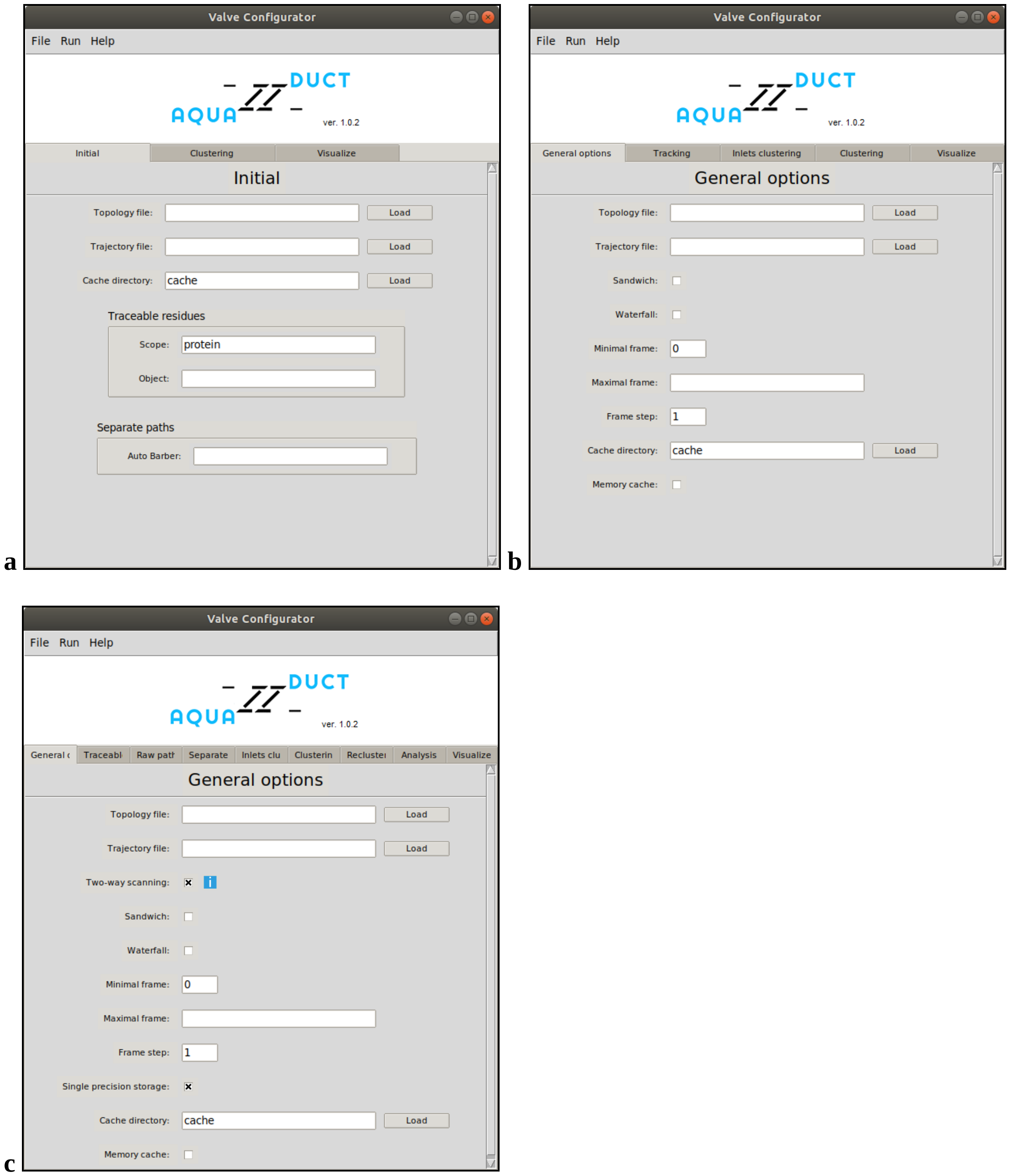

Supplementary Figure 16. An example of *kraken* output file from analysis of sample data set. (a) Clusters size graph showing the size of all identified clusters [in inlets] divided into INCOMING and OUTGOING group. (b) Relative clusters flow showing the contribution of particular exits in the total flow of the ligands in time. (c) Molecule entry time distribution plot shows you the color-coded paths sorted by the time they have entered the *Scope*. (d) Cluster size graph illustrating the percentage value of the size of each cluster. (e) The intramolecular flow graph shows how many ligands have been exchanged between clusters and in what direction. (f – i) Plots showing changes in the *Scope* and *Object* area and volume. (j) Clusters area plot. (k) 2D plot of energy profile along selected path.

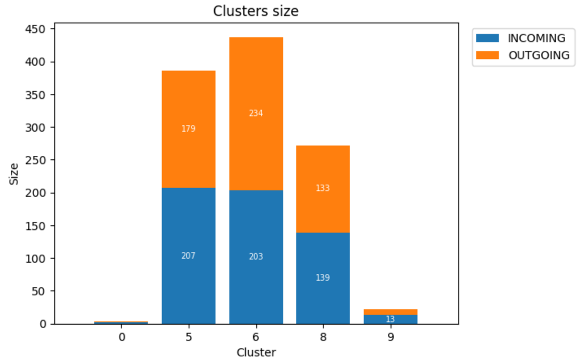

a

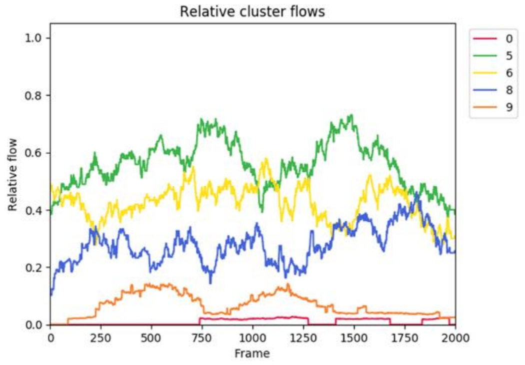

b

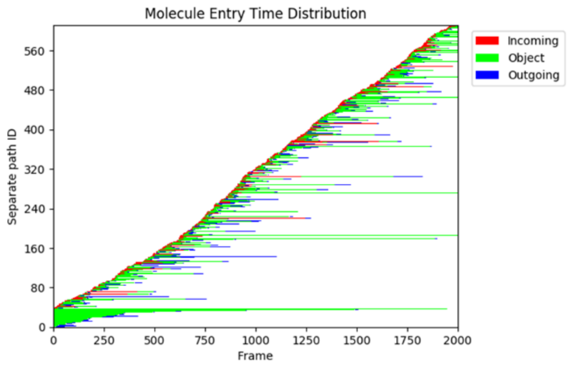

c

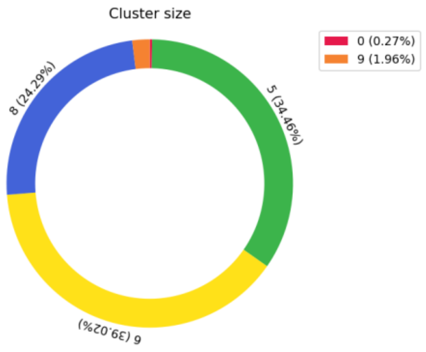

d

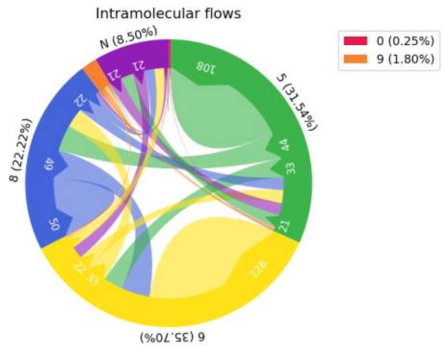

e

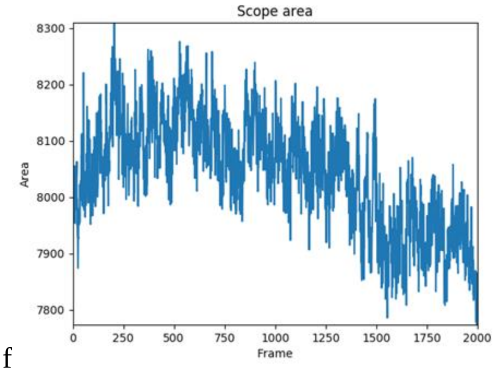

f

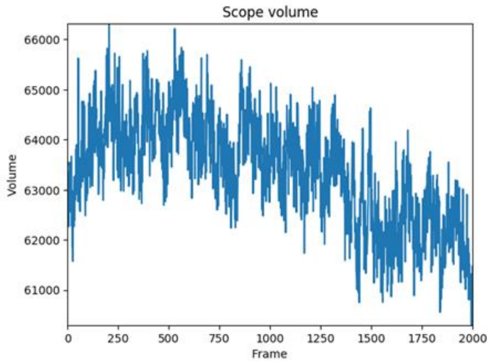

g

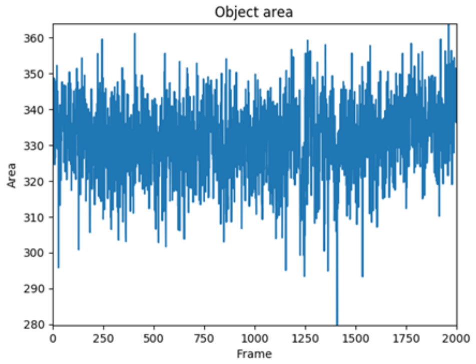

h

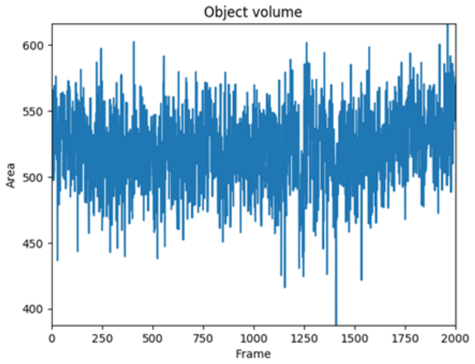
Object volume
Area
Frame
600
550
500
450
400
0
250
500
750
1000
1250
1500
1750
2000

i

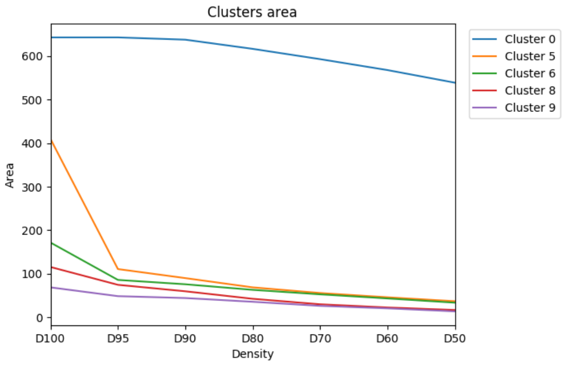
Clusters area
Area
Density
Cluster 0
Cluster 5
Cluster 6
Cluster 8
Cluster 9
600
500
400
300
200
100
0
D100
D95
D90
D80
D70
D60
D50

j

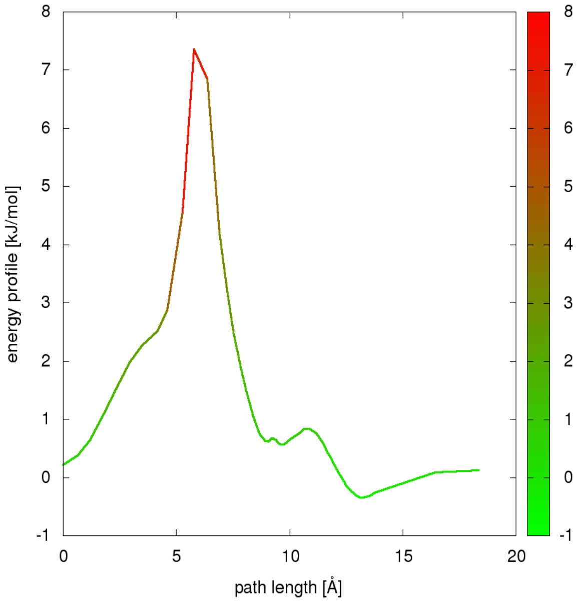
energy profile [kJ/mol]
path length [Å]
8
7
6
5
4
3
2
1
0
-1
0
5
10
15
20

k

Performance

The AQ performance was tested on a 500 ns simulations of C-terminal domain of msEH (PDB ID: 1CQZ; 4992 atoms immersed in 8488 molecules of water) and cytochrome CYP3A4b systems (PDB ID: 2V0M; 7758 atoms including a heme residue, immersed in 14733 molecules of water and 4 Cl^- ions mixture). Tests were run under Linux system, Intel Core i7 CPU @ 3.50GHz machine, 64 GB RAM and a 1TB NVMe SSD device.

AQ calculation time depends on two main factors: length of the MD simulation (measured by number of frames) and size of the *Object*. The latter factor controls number of traced molecules. The bigger the *Object* is, the more molecules are traced. The simulation length has an impact on the number of identified paths and their total length.

Following table shows AQ calculation time versus simulation length.

Table S1. AQ calculation time versus simulation length of tested systems.

Simulation length [ns]	msEH		CYP3A4b	
	**Number of identified paths**	**AQ calculation time [h]**	**Number of identified paths**	**AQ calculation time [h]**
**50**	561	2.30	222	2.14
**100**	759	6.26	442	4.82
**200**	1415	21.21	884	11.27
**500**	2915	99.35	2202	44.88

The correlation between total calculation time and total length of identified paths is close to linear (**Supplementary Fig. 17**).

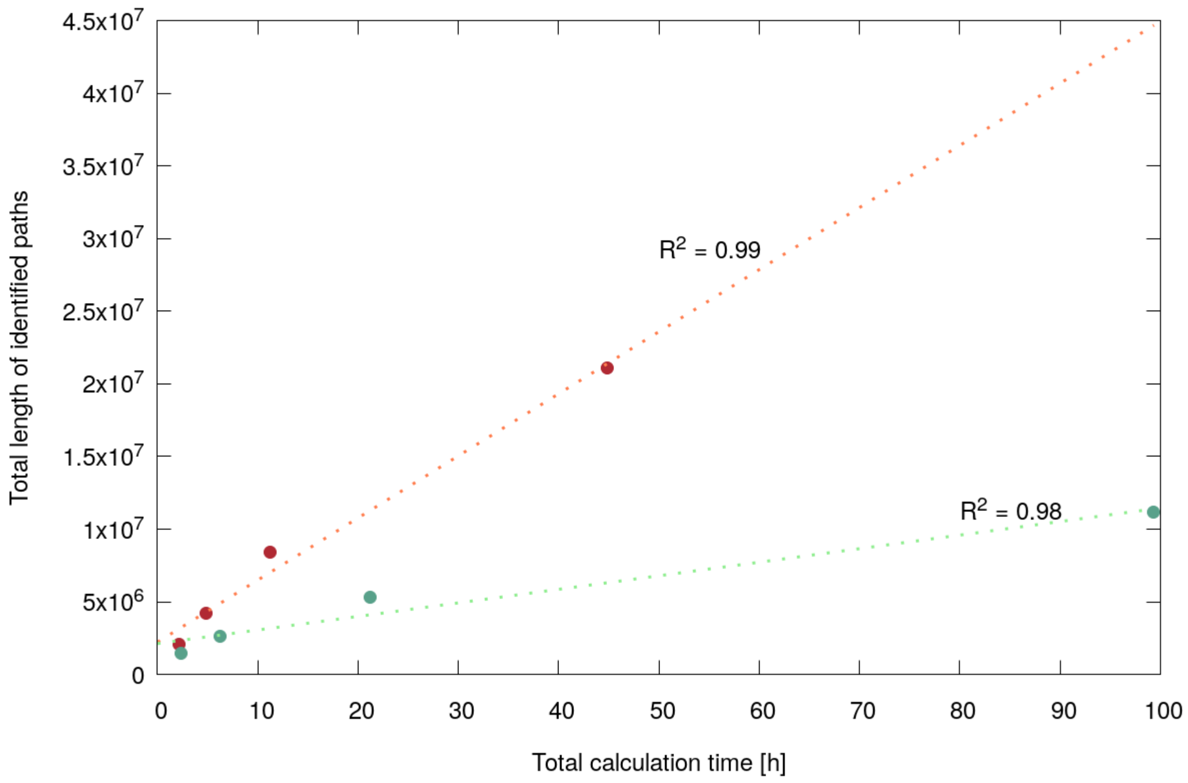

Supplementary Figure 17. The correlation between the total AQ calculation time in hours and the total length of identified paths in Å of two analyzed systems: cytochrome (red) and msEH (green).

The longer the simulation, the longer the identified paths. Depending on the system, the number of identified paths and their length impact the total length of AQ calculations. Based on the total calculation time of shorter simulations of the same system, the user can anticipate the calculation time of the longer simulation.

In case of longer simulations (more than 200 ns) or tracking of molecules bigger than water molecules (small ligands, (co)solvents), we recommend using lower sampling rate (i.e., a trajectory of snapshots saved every 2 or 5 picoseconds) or equivalent `step_frame` option. On **Supplementary Fig. 18** we showed the relationship between the total calculation time and the `step_frame` value on two analyzed systems.

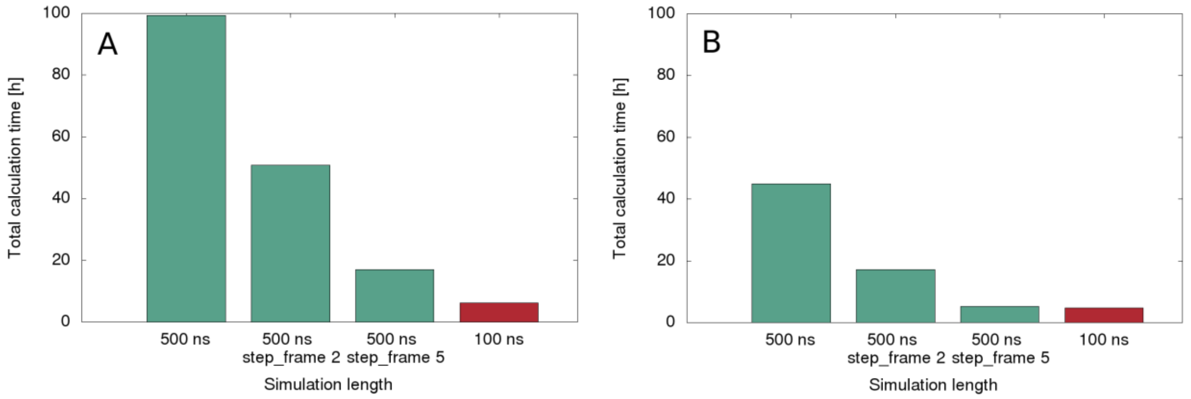

Supplementary Figure 18. The relationship between the total calculation time and the `step_frame` value of two systems: A) msEH, and B) CYP3A4b. The red box represents the total calculation time for a 100 ns simulation, for comparison.

References

Anandakrishnan,R. *et al.* (2012) H++ 3.0: Automating pK prediction and the preparation of biomolecular structures for atomistic molecular modeling and simulations. *Nucleic Acids Res*, **40**.

Antony,J. *et al.* (2000) Theoretical study of electron transfer between the photolyase catalytic cofactor FADH- and DNA thymine dimer. *J Am Chem Soc.*

Biedermannová,L. *et al.* (2012) A single mutation in a tunnel to the active site changes the mechanism and kinetics of product release in haloalkane dehalogenase LinB. *J Biol Chem*, **287**, 29062–29074.

Brezovsky,J. *et al.* (2016) Engineering a de Novo Transport Tunnel. *ACS Catal*, **6**, 7597–7610.

Case,D.A. (2018) Amber 18. *Univ California, San Fr.*

Cerutti,D.S. *et al.* (2008) Simulations of a protein crystal: Explicit treatment of crystallization conditions links theory and experiment in the streptavidin-biotin complex. *Biochemistry*.

D.A. Case, V. Babin, J.T. Berryman, R.M. Betz, Q. Cai, D.S. Cerutti, T.E. Cheatham, III, T.A. Darden, R.E. Duke, H. Gohlke, A.W. Goetz, S. Gusarov, N. Homeyer, P. Janowski, J. Kaus, I. Kolossváry, A. Kovalenko, T.S. Lee, S. LeGrand, T. Luchko, R. Luo, B.,X.W. and P. a. K. (2014) Amber 14. *Univ California, San Fr.*

Fishelovitch,D. *et al.* (2010) How does the reductase help to regulate the catalytic cycle of cytochrome P450 3A4 using the conserved water channel? *J Phys Chem B*, **114**, 5964–5970.

Frisch,M.J. *et al.* (2010) Gaussian09 Revision D.01, Gaussian Inc. Wallingford CT. *Gaussian 09 Revis C01*.

Jones,E. *et al.* (2001) SciPy.org. *SciPy Open source Sci tools Python2*.

Kellogg,E.H. *et al.* (2011) Role of conformational sampling in computing mutation-induced changes in protein structure and stability. *Proteins Struct Funct Bioinforma*.

Kovalenko,A. *et al.* (2010) Three-dimensional molecular theory of solvation coupled with molecular

dynamics in amber. *J Chem Theory Comput*, **6**, 607–624.
Li,P. and Merz,K.M. (2014) Taking into account the ion-induced dipole interaction in the nonbonded model of ions. *J Chem Theory Comput*.
Magdziarz,T. *et al.* (2017) AQUA-DUCT: A ligands tracking tool. *Bioinformatics*, **33**, 2045–2046.
Maier,J.A. *et al.* (2015) ff14SB: Improving the Accuracy of Protein Side Chain and Backbone Parameters from ff99SB. *J Chem Theory Comput*, **11**, 3696–3713.
Martinez,L. *et al.* (2009) PACKMOL: A package for building initial configurations for molecular dynamics simulations. *J Comput Chem*.
Michaud-Agrawal,N. *et al.* (2011) MDAnalysis: A toolkit for the analysis of molecular dynamics simulations. *J Comput Chem*.
Mitusińska,K. *et al.* (2018) Exploring Solanum tuberosum Epoxide Hydrolase Internal Architecture by Water Molecules Tracking. *Biomolecules*, **8**, 143.
Newie,J. *et al.* (2017) Lipoxygenase 2 from Cyanothece sp. controls dioxygen insertion by steric shielding and substrate fixation. *Sci Rep*.
Pedregosa FABIANPEDREGOSA,F. *et al.* (2011) Scikitlearn: Machine Learning in Python Gaël Varoquaux. *J Mach Learn Res*.
Rao,S. *et al.* (2017) A BEST example of channel structure annotation by molecular simulation. *Channels*.
Rosini,E. *et al.* (2011) On the reaction of d-amino acid oxidase with dioxygen: O 2 diffusion pathways and enhancement of reactivity. *FEBS J*, **278**, 482–492.
Royal Society of Chemistry (2015) ChemSpider. Search and Share Chemistry. *R Soc Chem*.
Saam,J. *et al.* (2010) O2 reactivity of flavoproteins: Dynamic access of dioxygen to the active site and role of a H+ relay system in D-amino acid oxidase. *J Biol Chem*, **285**, 24439–24446.
Sali,A. and Blundell,T.L. (1993) [Pmine5]Comparative protein modelling by satisfaction of spatial restraints. *J Mol Biol*.
Savitzky,A. and Golay,M.J.E. (1964) Smoothing and Differentiation of Data by Simplified Least Squares Procedures. *Anal Chem*.
Schrödinger (2015) The PyMOL Molecular Graphics System. *Schrödinger LLC wwwpymolorg*.
Serrano-Hervás,E. *et al.* (2018) Epoxide Hydrolase Conformational Heterogeneity for the Resolution of Bulky Pharmacologically Relevant Epoxide Substrates. *Chem - A Eur J*, **24**, 12254–12258.
Shahrokh,K. *et al.* (2012) Quantum mechanically derived AMBER-compatible heme parameters for various states of the cytochrome P450 catalytic cycle. *J Comput Chem*.
Sindhikara,D.J. *et al.* (2012) Placevent: An algorithm for prediction of explicit solvent atom distribution-Application to HIV-1 protease and F-ATP synthase. *J Comput Chem*, **33**, 1536–1543.
Stepankova,V. *et al.* (2013) Organic co-solvents affect activity, stability and enantioselectivity of haloalkane dehalogenases. *Biotechnol J*.
Subramanian,K. *et al.* (2018) Modulating D-amino acid oxidase (DAAO) substrate specificity through facilitated solvent access. *PLoS One*, **13**, e0198990.
Vanquelef,E. *et al.* (2011) R.E.D. Server: A web service for deriving RESP and ESP charges and building force field libraries for new molecules and molecular fragments. *Nucleic Acids Res*.
